# Multiphase Chemistry and Phase State Explain Nonlinear Effects in the Formation and Evaporation of SOA from Mixed Monoterpene Precursors

**DOI:** 10.1021/acsestair.5c00438

**Published:** 2026-04-08

**Authors:** Hyun Gu Kang, Masayuki Takeuchi, Nga L. Ng, Ulrich Pöschl, Thomas Berkemeier

**Affiliations:** † Multiphase Chemistry Department, 28309 Max Planck Institute for Chemistry 55218 Mainz, Germany; ‡ School of Civil and Environmental Engineering, Georgia Institute of Technology, Atlanta, Georgia 30332, United States of America; § School of Chemical and Bimolecular Engineering, Georgia Institute of Technology, Atlanta, Georgia 30332, United States of America; ∥ School of Earth and Atmospheric Sciences, Georgia Institute of Technology, Atlanta, Georgia 30332, United States of America

**Keywords:** organic aerosol, diffusion, partitioning, viscosity, oligomerization

## Abstract

A large fraction of airborne particulate matter consists of secondary organic aerosol (SOA), which can be highly viscous, leading to kinetic limitations in gas–particle partitioning and chemical reactivity. The underlying processes, however, have not been fully resolved and quantified in computational models. We use experimental data and the kinetic multilayer model of multiphase chemistry (KM3C) to investigate SOA formation by oxidation of limonene and α-pinene with the nitrate radical (NO_3_). The model explicitly treats gas–wall loss, gas–particle partitioning, as well as gas- and particle-phase chemistry, and includes a novel method for parametrizing bulk diffusivity from particle composition. KM3C utilizes and reproduces the temporal evolution of particle mass and thermal desorption mass spectrometry data (FIGAERO-CIMS) obtained in chamber experiments. The model can explain the observed slow evaporation of limonene SOA through slow bulk diffusivity and predicts the formation of a viscous surface crust. In mixed-precursor experiments, KM3C attributes nonadditive SOA mass yields to cross-reactions between α-pinene- and limonene-derived intermediates, leading to accretion products. We conclude that particle phase state, oligomerization, and multiprecursor effects have to be resolved to accurately describe and predict atmospheric SOA formation.

## Graphical abstract

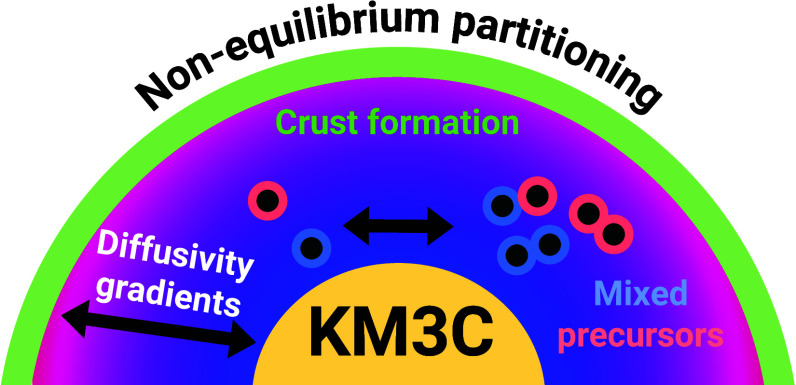

## 1 Introduction

Air quality models use absorptive partitioning theory to estimate organic aerosol (OA) formation.^1^ Donahue et al.^2^ built on absorptive partitioning theory to introduce the volatility basis set (VBS), which parametrizes secondary organic aerosol (SOA) formation by categorizing volatile organic compound (VOC) oxidation products by their vapor pressures. A VBS can be derived using SOA growth data from oxidation reactors or thermal desorption data from instruments such as the filter inlet for gases and aerosols chemical ionization mass spectrometer (FIGAERO-CIMS).^3,4^ While absorptive partitioning theory assumes rapid gas and particle equilibria and homogeneous particles,^1^ particles from experiments and ambient air can be viscous and may not reach equilibrium with the surrounding gas phase within their atmospheric lifetimes.^5–7^

Berkemeier et al.^8^ reacted α-pinene and limonene with the nitrate radical (NO_3_) in the Georgia Tech Environmental Chamber (GTEC), which can be temperature-controlled between 5 and 42 °C. They derived the oxidation product volatility distribution using the SOA growth and evaporation data with a kinetic model. However, the model considered only data from a scanning mobility particle sizer (SMPS), and the effects of a viscous particle phase state were only explored in a sensitivity study. As a consequence, the model required rapid and near-complete oligomerization of the limonene SOA to fit the experimental data. Schobesberger et al.^3^ developed a model to calculate FIGAERO-CIMS thermograms from volatility but assumed that the particle bulk is well-mixed. Here, we describe SOA growth and evaporation in a reaction chamber and parallel thermal desorption measurements with FIGAERO-CIMS using a single kinetic multilayer model and a single kinetic parameter set and explicitly account for the evolution of the particle phase state over time.

Typical accretion reactions in the particle phase of SOA are peroxyhemiacetal formation from hydroperoxides and carbonyls, esterification from alcohols and carboxylic acids,^9–11^ and acid-catalyzed aldol condensation.^12,13^ Bakker-Arkema and Ziemann^9^ mixed extracted SOA from α-pinene + O_3_ with a hydroperoxide probe molecule and found that the oligomerization equilibrium was reached within an hour. However, there are remaining uncertainties in both oligomerization rates and equilibrium constants, and a wide range of oligomer mass fractions have been reported, such as >50% for α-pinene + O_3_ SOA.^14,15^

Kinetic multilayer models can account for how bulk diffusivity (*D*_b_) affects chemistry^16–18^ and partitioning.^19,20^ In such models, the particle bulk is divided into layers in the form of spherical shells, which can exchange mass through diffusional transport. Kinetic multilayer models have been used previously to study the effects of diffusivity in SOA growth and evaporation,^8,20–25^ in addition to related applications such as the cloud formation ability,^19^ oxidative aging,^18,26–28^ and photochemical aging of OA.^16,29,30^ This multilayer bulk structure better explains scenarios where rapid mixing of the particle bulk cannot be assumed,^31–33^ but the computational expense of the model increases steeply with the number of bulk layers. Especially for SOA formation chemistry, where hundreds of chemical reactions and oxidation products play a role, a multilayer treatment of aerosol particles can quickly become infeasible.

VBS-based chemical mechanisms can help reduce the number of chemical compounds that must be considered when modeling the formation of SOA. VBS approaches often presume that each precursor VOC oxidizes to form products independent of the presence of other VOCs, thus assuming that the total SOA mass yield (*Y*_SOA_) is the sum of the yields of the individual precursors.^34^ However, experiments have found *Y*_SOA_ to not be a linear sum.^8,35–37^ Takeuchi et al.^36^ used FIGAERO-CIMS thermograms and *Y*_SOA_ collected during chamber experiments similar to those described in Berkemeier et al.^8^ to conclude that reactions between the condensed α-pinene and limonene oxidation products lead to nonadditive *Y*_SOA_. The authors proposed that cross-reactions between gas-phase intermediates or particle-phase products of the two monoterpenes may cause the nonlinearity, but such chemistry was not explicitly modeled.

The oxidation of gaseous species tends to reduce the vapor pressure of these species by adding functional groups onto their molecular backbones.^38^ In a VBS-based model, such oxidative aging can be reflected through shifts in the volatility distribution toward products with lower effective saturation mass concentrations (C*).^2,39^ This “volatility bin-hopping” has been used to model the shift in the volatility distribution of siloxane oxidation products^40^ and oil sand emissions^41^ by photochemical aging.

In this study, we use the kinetic multilayer model for multiphase chemistry (KM3C) to model the SOA growth and evaporation data from Berkemeier et al.^8^ and the FIGAERO-CIMS thermograms reported in Takeuchi et al.^36^ with a single chemical mechanism and a set of kinetic model parameters. We explore the effects of mixed-precursor dimer production from gas-phase peroxy radical reactions (RO_2_ + RO_2_), oligomerization in the particle bulk, gas-phase NO_3_ aging, and composition-dependent diffusivity. The Monte Carlo genetic algorithm (MCGA)^42^ is used to derive optimal model parameters from the experimental data.

## 2 Methods

### 2.1 Experiments

Details on the experiments conducted at the Georgia Tech Environmental Chamber (GTEC) facility are described in Berkemeier et al.^8^ and Takeuchi et al.^36^ In brief, α-pinene and/or limonene reacted with NO_3_ via molar excess injections of N_2_O_5_ (4:1 of N_2_O_5_ to double bond) into a dry and dark Teflon chamber in the presence of ammonium sulfate seed particles. Since α-pinene has lower *Y*_SOA_ than limonene when reacted with NO_3_, experiments were performed with higher concentrations of α-pinene than those of limonene to achieve similar SOA mass loadings.

Experiments started at a temperature of about 5 °C that increased to 42 °C in two steps over the course of hours. A scanning mobility particle sizer (SMPS) was used to monitor SOA growth and evaporation, and the data was adjusted for particle–wall loss.^43^ Four types of experiments were conducted: two where each monoterpene was oxidized separately (APN and LIM), one where both monoterpenes were oxidized together (MIX), and one sequential experiment where limonene was oxidized after α-pinene (SEQ).

A filter inlet for gases and aerosols chemical ionization mass spectrometer (FIGAERO-CIMS) was used to collect and thermally desorb particles on a Teflon filter in intervals of 60 min throughout the course of the experiment. We use a FIGAERO-CIMS thermogram from each chamber temperature set point (5, 25, and 42 °C), which is three thermograms for each experiment, for kinetic modeling.

### 2.2 Kinetic Multilayer Model for Multiphase Chemistry (KM3C)

The kinetic multilayer model for multiphase chemistry (KM3C) explicitly describes the temporal evolution of a multiphase chemical system by solving ordinary differential equations (ODEs) that contain transport fluxes and rates of chemical production and losses. The differential equations were solved using Matlab software (ode15s solver). The model builds on the earlier work by Shiraiwa and co-workers.^32,44^ The model code is automatically generated using the kinetic multiphase meta-model (KM-MEMO) code generator, which is a Matlab package program developed in-house to produce model files based on user inputs without having to manually compose the model code.^30^

We build on the model from Berkemeier et al.,^8^ where the previous model assumed that particles are well-mixed, by having multiple bulk layers to account for *D*_b_. We also incorporate adaptive layer resizing and moving–boundary algorithms, which allow for the particles to grow and shrink dynamically while avoiding numerical stiffness. As for the model compartment structure, we follow the one in Berkemeier et al.,^8^ which subdivided a reaction chamber into the wall, gas-phase, and particle-phase compartments. Thin gas layers surrounding the chamber wall and the particle bulk allow for the treatment of gas diffusion. Gas–wall loss is treated following Huang et al.^45^ Separate chemical mechanisms are employed in the gas and particle phases. The particle bulk was further subdivided into thin diffusion layers.

The depth of the near-surface particle bulk layer increases and decreases as the organic species adsorb and desorb. When the near-surface bulk layer becomes very thin due to evaporation, numerical stiffness increases sharply, which often leads to failure of the numerical ODE solver. We address this issue by adopting an adaptive layer resizing algorithm (Figure 1b), which merges adjacent bulk layers when the volume of a layer falls below a lower threshold. Likewise, the algorithm splits a layer when it exceeds an upper volume threshold, resulting in a dynamic number of bulk layers.

**Figure 1:**
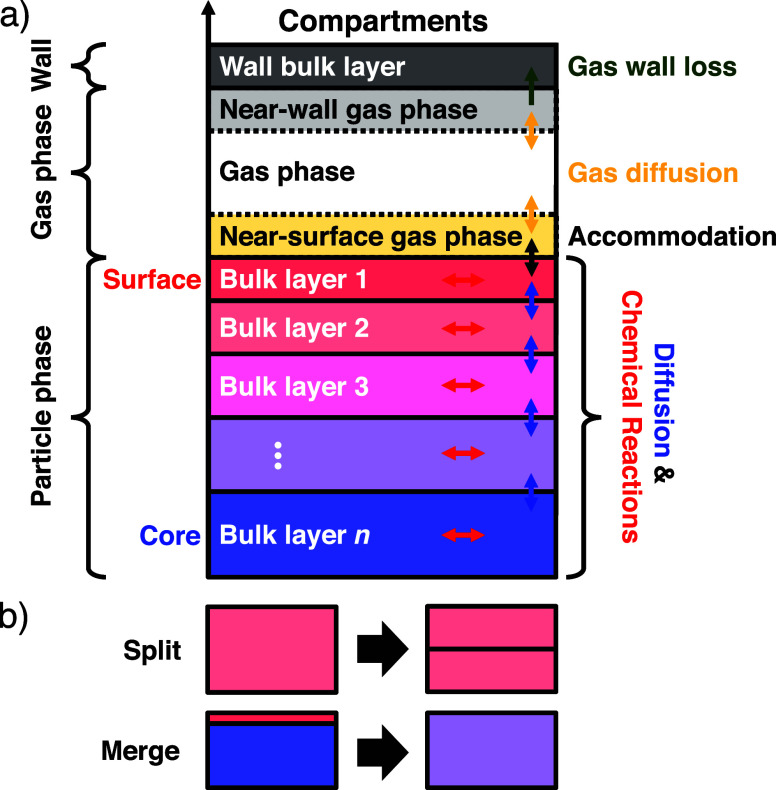
(a) Compartments and processes in the kinetic multilayer model KM3C and (b) illustration of the adaptive layer resizing scheme. KM3C subdivides a reaction chamber into the wall, gas, and particle phase compartments and models mass transport between and reactions inside each model layer. Particle-phase bulk layers split or merge as they exceed upper or lower size thresholds, respectively, leading to a dynamic number of layers throughout the calculation.

Note that the merging of bulk layers can lead to numerical diffusion,^46^ i.e., increased transport of matter compared to the real physical system, if concentrations in neighboring layers are different. The impact of numerical diffusion can generally be minimized by using a sufficient number of bulk layers so that concentrations between neighboring layers differ only slightly. The systems investigated in this study, however, exhibit steep concentration gradients at the particle surface, and merging of the topmost into the second bulk layer then leads to a sudden increase in evaporation of volatile species that were trapped under the near-surface layer. To resolve this issue, we incorporate a moving-boundary algorithm, similar to that used by Couvidat and Sartelet,^46^ where the volume of the near-surface layer does not shrink as a result of net desorption at the surface (Figures S1 and S2). The moving-boundary algorithm is described in detail in Section S1.

KM3C is also used to model thermal desorption in FIGAERO-CIMS explicitly. The model runs are initiated with the exact composition of particles (including internal diffusion gradients) from the chamber model runs, taken at a specific experimental time that coincides with the sampling interval of the FIGAERO-CIMS thermogram. The thermal desorption behavior of the particles when flowing pure nitrogen gas into the FIGAERO device is then simulated. KM-MEMO is also equipped with parameter optimization algorithms, including the Monte Carlo Genetic Algorithm (MCGA),^42^ which is used in this study to obtain globally fitting kinetic parameter sets (Section S4) using the chamber experiment bulk mass time series and the FIGAERO thermograms. In the first step, we use MCGA to optimize all kinetic parameters necessary to describe the pure precursor systems on the respective experimental data (APN and LIM). The parameters exclusive to the mixed precursor systems (i.e., properties of heterodimers) are optimized in a second optimization step only to the mixed precursor experimental data (MIX, SEQ). A sensitivity study of the optimized model parameters is shown and discussed in Section S5.

#### 2.2.1 Chemical Mechanism

The reaction mechanism used in this study builds on the mechanism presented in Berkemeier et al.,^8^ which was based on Master Chemical Mechanism v3 (MCM).^47^ The semiexplicit mechanism bins intermediates and oxidation products into categories based on molecular structural features (e.g., alkoxy radicals RO, peroxy radicals RO_2_) and volatility, respectively. We simplify aspects of the Berkemeier et al.^8^ mechanism by removing the distinction between the non-nitrated and nitrated organic species. Instead, all products are distributed across a single volatility basis set (VBS), which significantly reduces the number of model parameters and calculation time. We set logarithmically spaced volatility bins for condensing monomers in the range $C_{298}^{*}=1\times 10^{-4}-1\times 10^{3}\mu$ g m^–3^, where *C*_298_^*^ is the effective saturation mass concentration at 298 K.^2^ We also assign a separate bin for high-volatile species that do not partition. The C* are adjusted for temperature following Epstein et al.^48^ From the FIGAERO-CIMS data, we find that a large fraction (>80%) of detected ions in each experiment contain nitrogen (Figure S16) and so use a single VBS for nitrated and non-nitrated chemical species.

The precursor VOCs react with NO_3_ to form peroxy radicals (RO_2_), which undergo further radical chemistry until they eventually terminate into stable oxidation products that are represented by VBS species. Specific details are available in Section S3, and a schematic of the mechanism is shown in Figure S3. The mechanism also includes dimer formation through RO_2,apn_ + RO_2,lim_ → ROOR_het_ reactions, where the volatility bin branching is linearly interpolated and the *C*_298_^*^ of ROOR_het_ is optimized for between those of ROOR_apn_ and ROOR_lim_. To address potential nonlinear effects in heterodimer formation, we include a fit adjustment factor (γ_RO2+RO2_) for the RO_2,apn_ + RO_2,lim_ reaction, eq 1. We set the bounds of γ_RO2+RO2_ to be within an order of magnitude of *k*_RO2+RO2,lim_, which covers the range between *k*_RO2+RO2,apn_ and *k*_RO2+RO2,lim_ and reflects the wide range of *k*_RO2+RO2_ reported in the literature.^49–51^

1 $$k_{RO2+RO2,het}=k_{RO2+RO2,\lim}\times \gamma_{RO2+RO2}$$

The gas-phase chemical mechanism includes aging of oxidation products with NO_3_ by the volatility bin assigned to them. We assume that hydrogen abstraction by NO_3_ adds a functional group, such as –OH or–OOH, to the molecule and reduces the molecule’s *C*_298_^*^ by 2 orders of magnitude.^38^ We optimize for the NO_3_-aging rate coefficient (*k*_aging_) for each monoterpene precursor in a range consistent with aliphatic species.^52,53^

In the particle phase, oxidation products can react to form oligomers, which we presume do not evaporate. Following Berkemeier et al.,^8^ we assume reverse oligomerization to occur as first-order reactions and the forward oligomerization as second-order reactions by multiplying a first-order rate coefficient with the mass fraction of monomeric reaction partners in a given model layer (*M*_f_). We fit the forward and reverse oligomerization rate coefficients (*k*_fwd,olig_ and *k*_rev,olig_) and energies of activation (*E*_a,fwd_ and *E*_a,rev_). Oligomers decompose into the original monomers, and the mechanism includes reactions between α-pinene and limonene monomers to form particle-phase hetero-oligomers. The rate coefficients for the hetero-oligomer formation and dissociation are assumed to lie between the values for the pure α-pinene and limonene systems (eqs 2 and 3), which we denote as “scale” in the below equations. We include a fitted adjustment factor (γ_fwd,olig_ and γ_rev,olig_) to the rate coefficients to address nonlinear effects.

2 $$k_{fwd,olig,het}=\gamma_{fwd,olig}\times scale\left(k_{fwd,olig,apn},k_{fwd,olig,\lim}\right)\times M_{f}\times \exp\left(\frac{E_{a,fwd,het}}{R}\times \left(\frac{1}{298}-\frac{1}{T}\right)\right)$$

3 $$k_{rev,olig,het}=\gamma_{rev,olig}\times scale\left(k_{rev,olig,apn},k_{rev,olig,\lim}\right)\times \exp\left(\frac{E_{a,rev,het}}{R}\times \left(\frac{1}{298}-\frac{1}{T}\right)\right)$$

#### 2.2.2 Composition-Dependent Bulk Diffusivity (*D*_b_)

Kinetic multilayer models allow assigning distinct *D*_b_ for every model layer based on molecular composition.^16,19^ A gradient of diffusivity based on composition has been proposed for systems analogous to SOA,^54,55^ and SOA with higher fractions of low-volatiles have been observed to be more viscous.^56^

Previous studies found a log–log relationship between vapor pressure and viscosity, where less volatile species tend to have higher viscosities.^57–61^ We thus prescribe a linear relationship between the decadal logarithms of the effective saturation mass concentration of species *i* at 298 K, *C*_298,i_^*^, and the self-diffusivity at 298 K, *D*_b,298,*i*_.

4 $$\log10\left(D_{b,298,i}\right)=m\times \log10\left(C_{298,i}^{*}\right)+b$$

The slope (*m*) and intercept (*b*) of this log–log relationship (eq 4) are obtained by fitting to the experimental data, and we use the same relationship to calculate the *D*_b,298,*i*_ of the monomers and dimers (Figure S4). As oligomers are nonvolatile in this model, they are assumed to contribute with a *D*_b,298,*i*_ that is a fraction *x*_olig_ of the *D*_b,298,*i*_ from the lowest volatility bin of the model.

To calculate the diffusivity of a given bulk layer *l* at 298 K (*D*_b,298,*l*_) from the composition of that bulk layer, we use the Vignes equation^18,62^ (eq 5), which is a logarithmic mixing rule that weighs *D*_b,298,*i*_ with the molar fraction *x*_*i*,*l*_ of species *i* inside layer *l*.

5 $$D_{b,298,l}=\prod {\left(D_{b,298,i}\right)}^{x_{i,l}}$$

We adjust *D*_b_ for temperature with the Clausius–Clapeyron eq (eq 6), where Δ*H* ^dif^ is the effective enthalpy of diffusion and *R* is the gas constant. We fit Δ*H* ^dif^ between 50 and 150 kJ mol^–1^, a range consistent with that of water–sucrose solutions.^63^

6 $$D_{b,l}=D_{b,298,l}\times \exp\left(-\frac{\Delta H^{dif}}{R}\left(\frac{1}{T}-\frac{1}{298}\right)\right)$$

## 3 Results and Discussion

### 3.1 Phase State Effects on SOA Growth and Evaporation

The time series of SOA mass concentrations in the pure precursor experiments (LIM, APN) is well-captured by the kinetic model, including the evaporation upon increase in chamber temperature (Figure 2a,b). The APN experiment shows sensitivity to *k*_aging_ due to the higher percentage of products of higher volatility (Figure S12); the model is sensitive to the monomer volatility distributions in both, APN and LIM, experiments (Figure S10). While Berkemeier et al.^8^ also achieved a good fit with the SOA mass, the previous model did so with a rapid and high degree of oligomerization. Here, the optimized model has a lower degree of oligomerization. The formation and evaporation occur more gradually for limonene SOA than for α-pinene SOA. The model finds that the bulk diffusivity in the APN experiment (Figure 2d) is initially higher than in the LIM experiment (Figure 2c) and that evaporation of α-pinene SOA is initially not diffusion-limited. In both experiments, diffusivity in the particle phase decreases with time as semivolatiles evaporate. Especially, the *D*_b_ of the near-surface bulk layer becomes suppressed compared to the underlying layers upon an increase in chamber temperature. The model suggests this surface crust forms by accumulation of low-volatile species and nonpartitioning oligomers at the surface (Figure 2e,f). A viscous surface crust (*D*_b_ ≈ 1 × 10^–17^ cm^2^ s^–1^) forms upon the first increase in chamber temperature to 25 °C for the LIM experiment and upon the second increase in chamber temperature to 42 °C in the APN experiment, each time slowing evaporation considerably. The formation of the crust in APN at higher temperatures coincides with oligomerization and the evaporation of more volatile species. These crusts delay the evaporation of underlying species, similar to the diffusion-limited evaporation found in polymer–solvent systems.^55^

**Figure 2:**
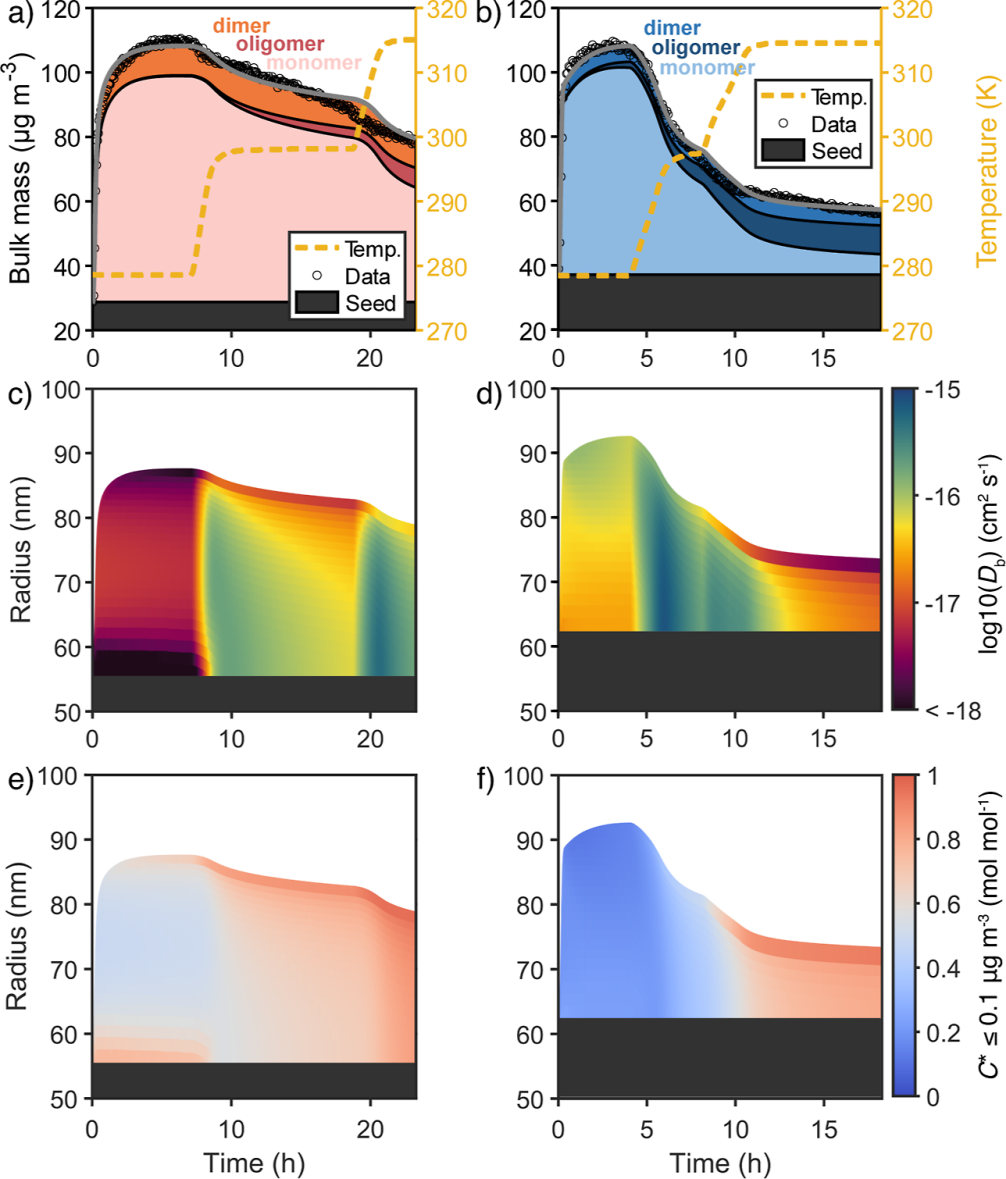
SOA mass concentrations and model results for bulk composition in the chamber for LIM (left column) and APN experiments (right column). Panels (a,b) show the time evolution of SOA mass concentrations obtained from SMPS data (markers) and the model (gray line). Subdivisions of the filled area underneath the modeled SOA mass concentrations denote the contribution of monomers, gas-phase dimers, and particle-phase oligomers to the total mass. The temperature during the model runs is shown as an orange dashed line. Panels (c,d) use color-coding and subdivision of the filled area underneath the modeled SOA mass concentrations into individual model layers to show the composition-dependent *D*_b_ for each bulk layer. Likewise, panels (e,f) display the molar fraction of all low-volatile species and oligomers (*C*_298,i_^*^ ≤ 0.1 μg m^3^) in each bulk layer. The model shows surface crust formation for both pure monoterpene precursors, which can be accredited to the increased concentration of low-volatile species and oligomers at the particle surface.

Li et al.^64^ used EPISuite to calculate the glass transition temperatures and vapor pressures of organic compounds and provided an empirical relationship between glass transition temperature and vapor pressure (Section S6). The *D*_b_ – C* relationship (eq 4) is largely consistent with the data analyzed by Li et al. (2020) for the range of *C*_298_^*^ in this study and does not require an upper viscosity limit (Figure S11). However, the data set is sparse for low *C*_298_^*^ species, and KM3C is sensitive to the composition-dependent bulk diffusivity parameters (Section S5, Figure S15). Thus, further research is needed to better constrain the relationship between viscosity and volatility of organic compounds.

The model also fits the MIX and SEQ experiments with reasonable accuracy (Figure 3a,b), using the same kinetic model parameters as for the pure precursor experiments, APN and LIM. In the MIX experiment, the model finds that the surface crust forms early on (Figure 3c), like with LIM, and the distribution of α-pinene and limonene species throughout the particle is more homogeneous compared with that of SEQ (Figure S5). Note that we find α-pinene oxidation products to condense slightly before the monomeric limonene oxidation products due to the faster oxidation chemistry of α-pinene in the gas phase (Figure S5a,b). Furthermore, accretion products of oxidation products from both monoterpenes (hetero-oligomers) form efficiently, which titrate away the less numerous monomeric limonene oxidation products near the core of the particle (Figure 3e). For SEQ, the model finds that a more viscous limonene SOA phase forms on the top of the less viscous α-pinene SOA (Figure 3d) and that heterodimers do not initially form. At 5 °C, both phases hardly mix, as hypothesized by Boyd et al.,^65^ and this division persists until the chamber temperature is increased to 25 °C where mixing happens of both phases occurs (Figure 3f). Consequently, the more volatile α-pinene species are trapped under the limonene species at the start, and hetero-oligomers do not form until the particle mixes (Figure S5). Both systems show formation of a viscous surface crust upon heating.

**Figure 3:**
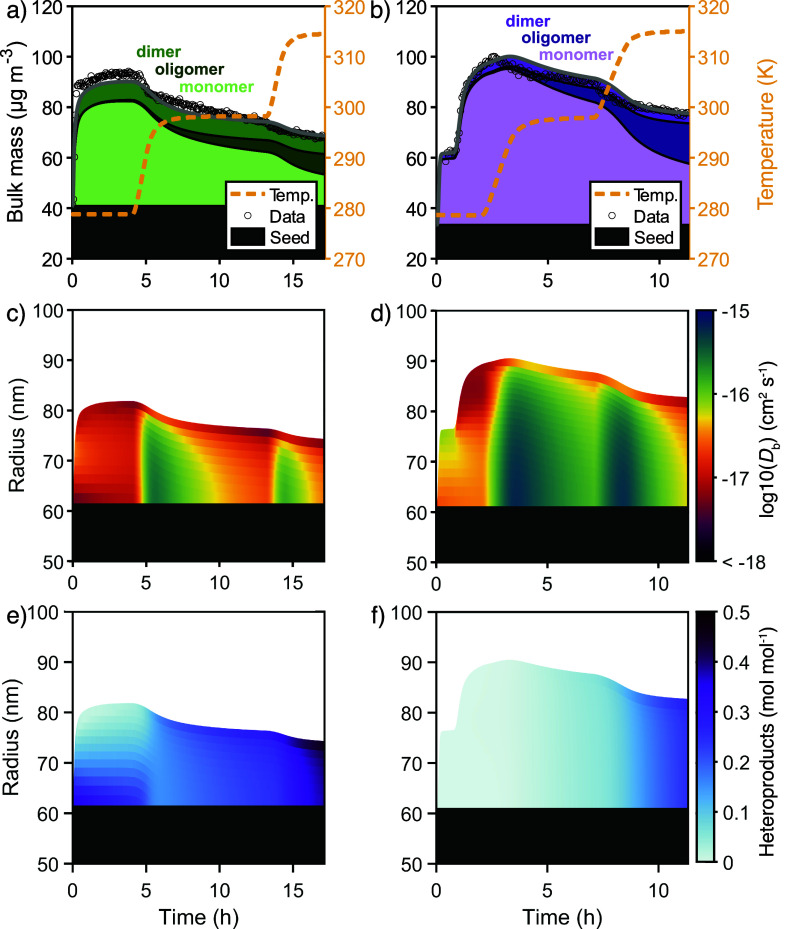
SOA mass concentrations and model results for bulk composition in the chamber for MIX (left column) and SEQ experiments (right column). Panels (a,b) show the time evolution of SOA mass concentrations obtained from SMPS data (markers) and the model (gray line). Subdivisions of the filled area underneath the modeled SOA mass concentrations denote the contribution of monomers, gas-phase dimers, and particle-phase oligomers to the total mass. The temperature during the model runs is shown as an orange dashed line. Panels (c,d) use color-coding and subdivision of the filled area underneath the modeled SOA mass concentrations into individual model layers to show the composition-dependent *D*_b_ for each bulk layer. The model slightly underpredicts *Y*_SOA_ in the MIX experiment. (e,f) show the sum of the molar fractions of heterodimers and hetero-oligomers.

To test the effect that slow bulk diffusion has on partitioning, we run the model with a single well-mixed bulk layer but otherwise the same model parameters (Figure 4 and Figure S6). The model is sensitive to both slope and intercept of the *D*_b_ – C* relationship (eq 4), where slower diffusion generally results in slower evaporation (Figure S11). In the case of LIM (Figure 4), gas–particle equilibrium is rapidly achieved in the monolayer model, not capturing the observed gradual decrease in particle mass. The SOA mass after 24 h, however, is very similar in both simulations. A monolayer model run for the APN experiment is fairly comparable with the result of the multilayer model (Figure S6), which is consistent with the earlier finding that evaporation of fresh α-pinene SOA is not diffusion-limited. However, we note crust formation in the model is a consequence of the *D*_b_ – C* relationship (eq 4), where a steeper slope would result in a less diffusive crust. In the absence of additional data, we cannot definitively assert whether slow bulk mixing or diffusivity gradient formation are unique solutions to explain the observed data. Nevertheless, diffusion-limited evaporation via crust formation is proposed in other particle systems^55^ and offers a plausible explanation for the slow SOA evaporation examined in this manuscript.

**Figure 4:**
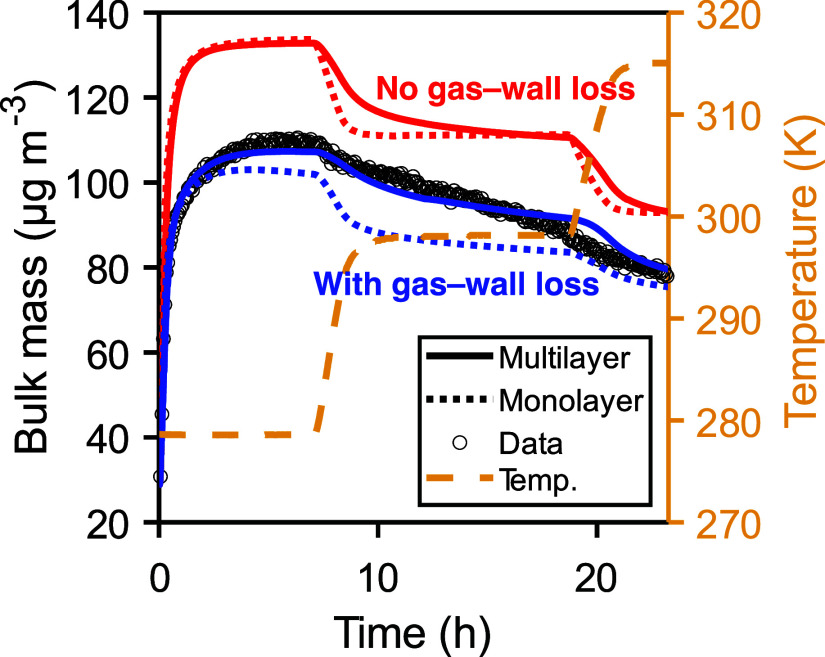
Effect of gas–wall loss and bulk diffusivity on SOA formation in LIM. Solid and dotted lines are results from the full multilayer model and a simplified monolayer version, respectively. When the particle is well-mixed in the monolayer model, less SOA is formed as repartitioning of species to the gas phase is facilitated, leading to gradual loss to the chamber walls. In the full multilayer model, slow bulk diffusivity can shield semivolatile species from being lost to the walls.

Furthermore, we find that peak *Y*_SOA_ is higher in the simulation with the multilayer model. This result is counterintuitive as semivolatile organics on the surface of a viscous particle may be incorporated more slowly into the particle bulk, which may result in slower SOA growth. As shown in Figure 4, there is more SOA formation in LIM without gas–wall loss (red solid and dotted lines), and there is no significant effect of bulk diffusion on peak *Y*_SOA_. The reason that slow bulk diffusion can increase *Y*_SOA_ in chamber experiments is that gas–particle repartitioning is slowed by bulk diffusion and thus semivolatile species are shielded from gas–wall loss, and similar results are found with the other scenarios (Figure S7). This result highlights the findings in McVay et al.,^66^ where both kinetically limited condensation and gas–wall loss time scales need to be considered when modeling SOA formation.

### 3.2 Nonadditivity of *Y*_SOA_

Figure 5 compares the SOA mass yield (*Y*_SOA_) obtained with the kinetic model to the data reported in Berkemeier et al.^8^ and in Takeuchi et al.^36^ We generate the yield curves by adjusting the initial VOC and N_2_O_5_ concentrations while keeping the ratio [VOC]/[N_2_O_5_] constant. We report total *Y*_SOA_ for all experiments. For the mixed precursor experiments (MIX, SEQ), we also report yields for the individual precursors, attributing the gas- and particle-phase heterodimers in equal parts to α-pinene and limonene.

**Figure 5:**
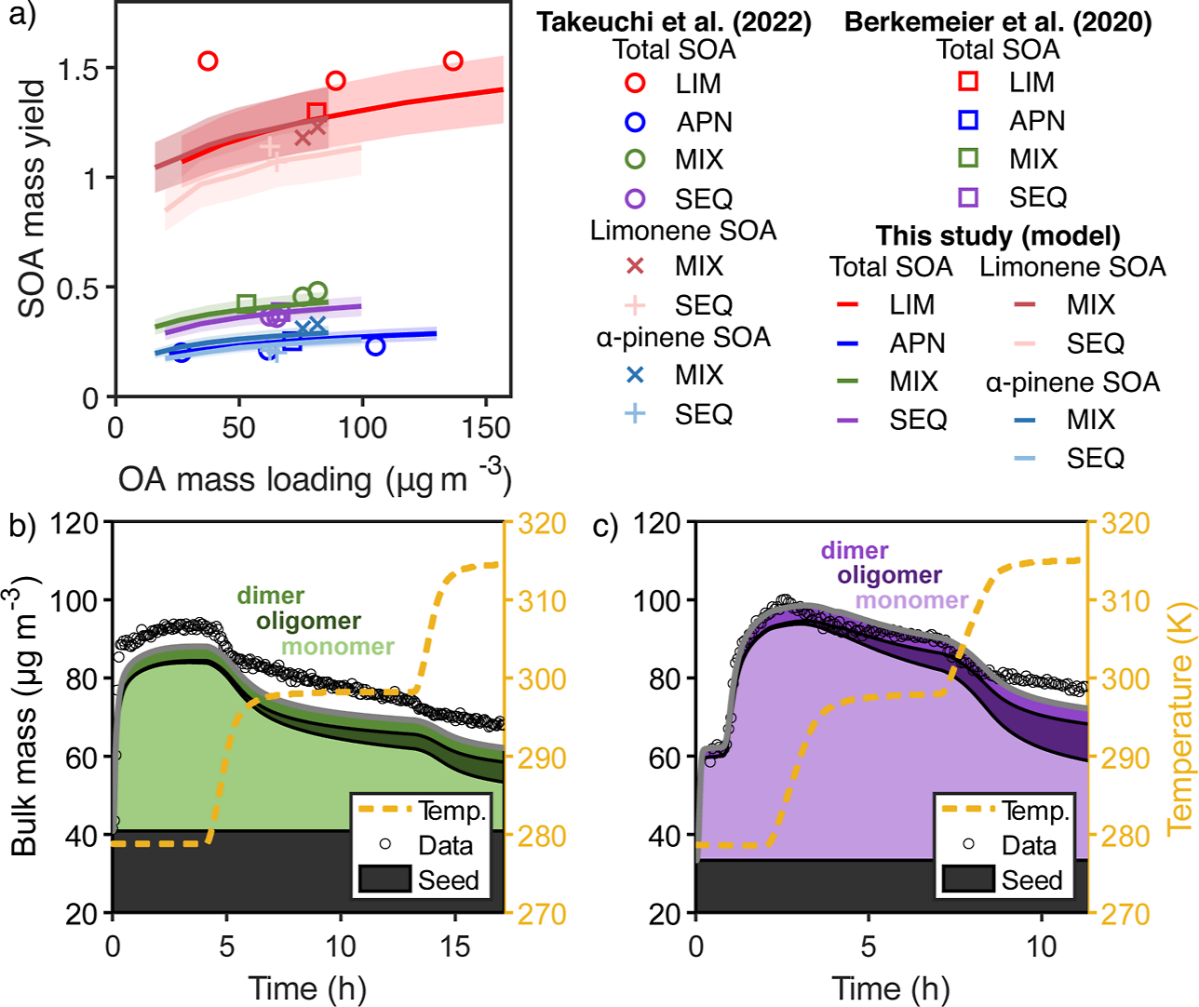
(a) SOA mass yield (*Y*_SOA_) curves (lines) obtained using KM3C and compared to measured values (markers) from Berkemeier et al.^8^ and Takeuchi et al.^36^ The shaded areas indicate the modeled uncertainty assuming ±10% error in the initial VOC and N_2_O_5_ concentrations. Total refers to *Y*_SOA_ calculated with the sum of α-pinene and limonene mass reacted. For SEQ and MIX, Takeuchi et al.^36^ used mass spectra to separate the α-pinene and limonene SOA, and we draw *Y*_SOA_ for each monoterpene. SOA mass concentrations in the chamber for (b) MIX and (c) SEQ experiments in modified model simulations in which the rate coefficient parameters for the heteroproduct formation reactions are linearly interpolated between those of α-pinene and limonene. For the MIX experiment, model results are notably lower than experimental measurements, which indicates nonlinear effects in the rate coefficients of cross-reactions.

When accounting for cross-reactions between α-pinene and limonene oxidation products to form heterodimers, we find that the modeled *Y*_SOA_ agrees well with the experimental results. The model reproduces a small enhancement in the SOA yield of α-pinene in the MIX experiment compared to that in the APN experiment, but the same enhancement is not found in the SEQ experiment (Figure 5a). The model suggests that mixing between the monoterpene products is initially limited in the SEQ experiment, causing a limonene SOA shell to form around an α-pinene core (Figure S5d,e). The spatial separation of oxidation products in the particle phase and the temporal separation of peroxy radicals in the gas phase leads to reduced formation of heterodimers from cross-reactions (Figure 3f). Previous studies^67,68^ suggested that incomplete mixing or phase separation may affect *Y*_SOA_ in multiprecursor experiments. Here, the model assumes full thermodynamic miscibility between limonene and α-pinene oxidation products, and spatial separation is only due to diffusion limitations in the particle phase.

The model returns a decrease in limonene SOA yield in the SEQ experiment (Figure 5a), in line with the experimental observations by Takeuchi et al.^36^ This is due to a shift in the product spectrum toward a lower abundance of gas-phase dimers in the particle phase (Figures 3b) caused by lower limonene precursor (and thus RO_2_) concentrations. Surprisingly, while limonene oxidation products do not fully mix into the existing α-pinene SOA particles, the viscous phase state does not reduce limonene SOA yield in the SEQ experiment (Figure S8a). This is because of the effect of shielding semivolatiles from gas–wall loss discussed for the LIM experiment above (Figure 4), which leads to an overall increase in SOA yield due to the viscous phase state. Note that the partitioning of low-volatile species, which almost fully condense, is hardly affected by particle phase state in the model.

When the heterogeneous reaction parameters are linearly interpolated between α-pinene and limonene, there is a marked reduction in the simulated SOA mass throughout the MIX experiment, while the SEQ experiment shows faster evaporation at the 42 °C temperature set point (Figure 5b,c). Thus, based on the global optimization results in this study (Section S4), we propose that the reaction rate coefficients of cross-reactions between α-pinene and limonene oxidation products must be faster than one would obtain from linear combination of the same reactions leading to the homodimers. The MCM assigns a *k*_RO2+RO2_ of 9.20 × 10^–14^ cm^3^ s^–1^ for the pure systems of α-pinene and limonene. The global model optimization in this study suggests an enhanced rate coefficient for the gas-phase cross-reaction, RO_2,apn_ + RO_2,lim_, of 7.18 × 10^–13^ cm^3^ s^–1^.

Reported RO_2_ + RO_2_ rate coefficients vary widely in the literature and depend on the molecular structures.^49,50^ Some of the enhancement in the rate coefficient we infer from the model results may be reflective of the differences in the structures between RO_2,apn_ and RO_2,lim_. For instance, Franzon et al.^51^ find that *k*_RO2+RO2_ for reactions involving an acyl peroxy radical can reach 10^–11^ cm^3^ s^–1^. The particle-phase oligomerization parameters are summarized in Table 1. The current optimized values suggest that the rate coefficients for the formation of heterodimers are more consistent with the slightly higher formation rate of α-pinene homodimers than with the formation rate of limonene homodimers. We find that the model is sensitive to heterodimer formation in the MIX experiment (Figure S14), where stronger formation of heterodimer results in stronger initial SOA growth. As a consequence, the MIX experiment shows a higher oligomer content than the LIM experiment.

**Table 1:** (a) Optimized First-Order Rate Coefficients (s^–1^) and Energies of Activation (kJ mol^–1^) for Forward and Reverse Oligomerization Reactions

	α-pinene	limonene	heterogeneous
*k*_fwd,olig_	1.11 × 10^–5^	3.00 × 10^–6^	9.52 × 10^–6^
*k*_rev,olig_	3.03 × 10^–6^	3.53 × 10^–5^	8.08 × 10^–7^
*E*_a,fwd_	55.0	98.6	98.4
*E*_a,rev_	42.1	44.2	43.1

In the ambient atmosphere, many VOCs are oxidized simultaneously; thus, atmospheric conditions likely resemble more the MIX experiment than the SEQ experiment. Nonlinearity of *Y*_SOA_ has been found in other VOC mixtures.^69,70^ As part of the nonadditivity arises from gas-phase dimer formation, future studies should look into the effects of RO_2_ fate^49^ on *Y*_SOA_ in precursor mixtures. Nevertheless, we find that *Y*_SOA_ nonlinearity is not only due to gas-phase kinetics but also due to the phase state of the particles as the formation of heterodimers can be limited by slow diffusion, as shown in the SEQ experiment. We conclude that particle phase state needs to be considered in order to accurately model the growth and fate of atmospheric SOA, which is particularly relevant in dry and cold environments.^7^ Although the relationship between bulk diffusivity and volatility presented in this study applies directly to SOA under dry conditions, the plasticizing effects of water, which increase diffusivity coefficients, must be considered at higher relative humidities.^6,71^ An approximate mathematical description has previously been achieved using the κ-Köhler theory and the Gordon–Taylor equation;^19,64,72,73^ however, a full description of the relationships of volatility, hygroscopicity, and diffusivity in SOA–water mixtures remains to be determined and is a subject of future studies.

### 3.3 Thermal Desorption

We use KM3C to model FIGAERO-CIMS sum thermograms based on the composition calculated during SOA growth and evaporation in the chamber experiment. The model is overall able to capture the FIGAERO-CIMS thermogram shapes and their temporal evolution (Figure 6). We show one thermogram from each temperature step, where the black, red, and blue colors correspond to the 5, 25, and 42 °C temperature set points in the chamber, respectively. Overall lower desorption temperatures in the thermogram for the APN experiment (Figure 6b) suggest that SOA is composed of more volatile species compared to SOA in the LIM experiment (Figure 6a). The thermogram distribution shifts to higher temperatures as the experiment progresses. While the APN thermogram exhibits a single peak, the LIM, MIX, and SEQ thermograms show two peaks. In the model, the first thermogram peak results from the desorption of semivolatile monomers and the second peak in the thermograms results from the evaporation of low-volatile monomers, dimers, and dissociating oligomers. When describing FIGAERO-CIMS data, the model is sensitive to the activation energies of the forward and reverse oligomerization reactions, *E*_a,fwd_ and *E*_a,rev_ (Figure S13), as oligomer decomposition is driven by these parameters at the higher temperatures of the FIGAERO. The first peak generally decreases relative to the second peak with every heating step, which is consistent with the model result that semivolatile monomers evaporate predominantly. Notably, α-pinene SOA shows pronounced particle-phase oligomerization in the model when heated to 42 °C, causing a shoulder of the first peak to develop. We note that different chemical species can have different sensitivities to the iodide CIMS,^74^ which is not accounted for in this study and may explain part of the discrepancy between the model and the experiment. For instance, Song et al.^75^ found that iodide CIMS sensitivity increases with the addition of polar functional groups. The addition of such functional groups onto a molecule decreases the molecule’s vapor pressure,^38^ and such low-volatile molecules likely desorb at higher temperatures in the FIGAERO. Accordingly, the second desorption peak seen in the LIM, MIX, and SEQ experiments may include a greater number of such highly functionalized molecules that the iodide CIMS is more sensitive to, which may skew the fitted model toward low-volatile species.

**Figure 6:**
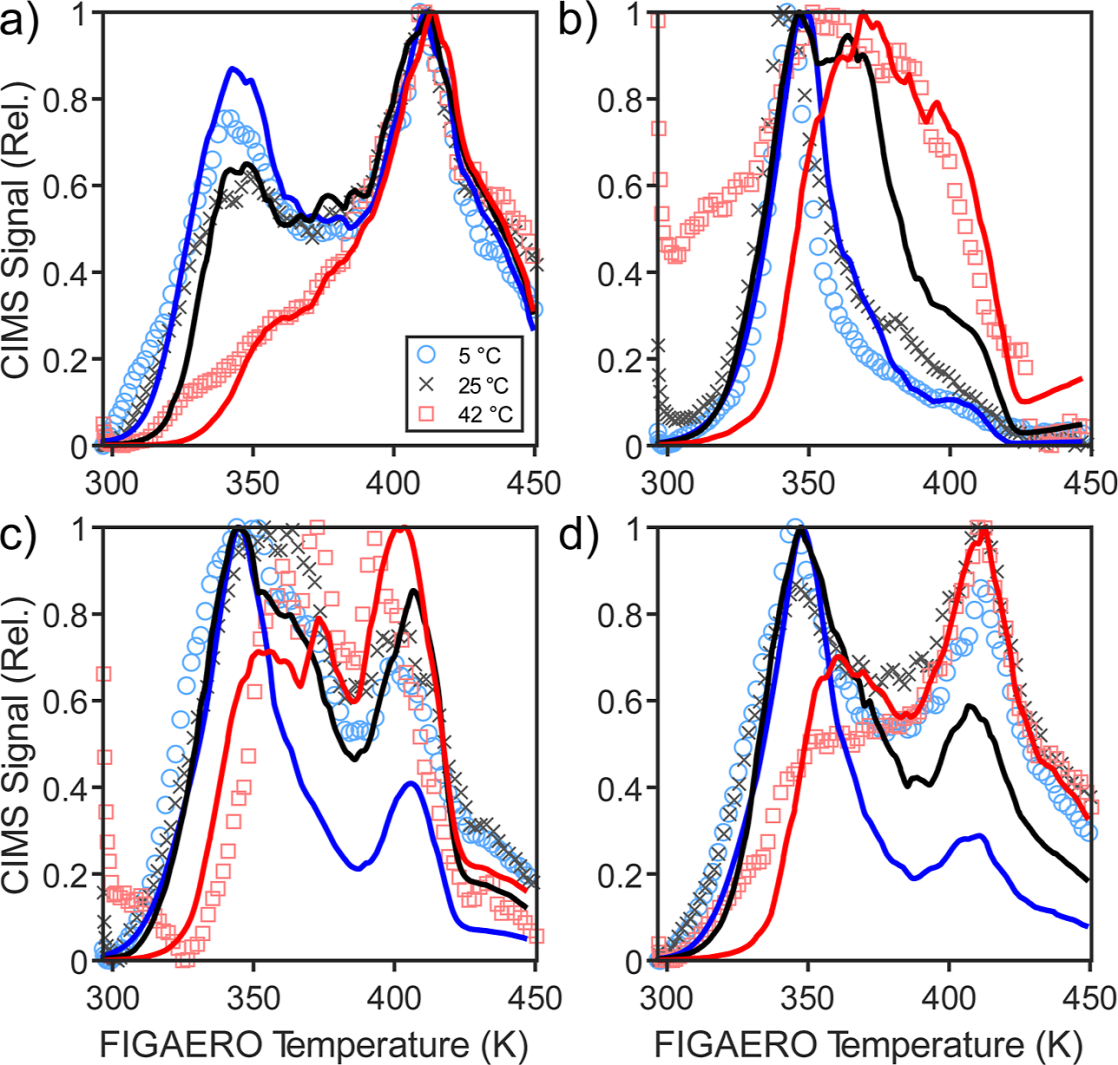
FIGAERO-CIMS thermograms of (a) LIM, (b) APN, (c) MIX, and (d) SEQ experiments. Lines and markers indicate the model and normalized data from the three temperature steps in the chamber experiment (5 °C: blue, 25 °C: black, 42 °C: red). The legend refers to the temperatures in the chamber during the sampling period.

While KM3C predicts a surface crust as a reason for slow evaporation in the LIM experiment, Berkemeier et al.^8^ achieved a good fit to the SOA mass data with a model that assumes a well-mixed particle bulk. This monolayer bulk model attributed the slow evaporation to the decomposition of particle-phase oligomers. When refitting KM3C with a single bulk layer, we reproduce this result from the earlier study (Figure S9a). However, the FIGAERO thermograms simulated with the particle composition from these well-mixed bulk calculations do not exhibit the double-peak behavior observed in the LIM experiment. Instead, the high degree of oligomerization results in a single peak desorbing at high temperatures (Figure S9b). Thus, the addition of FIGAERO-CIMS data adds a strong and important constraint on the model parameter space, further building evidence that the bulk diffusivity can have a pronounced effect on the evaporation of SOA. Furthermore, the KM3C model results show how slow bulk diffusion can affect the mechanistic interpretation of SOA evolution in chamber experiments. Here, KM3C attributes the slow evaporation of limonene SOA in the chamber to kinetically limited evaporation caused by formation of a viscous surface crust, while a model that was fitted to the data without accounting for slow bulk diffusion^8^ attributed the phenomenon to strong oligomer formation and decomposition.

In addition to laboratory chamber experiments, bulk diffusivity affects cloud formation and chemical fate in the atmosphere.^18,19^ Zaveri et al.^76^ studied urban plume measurements from aircraft campaigns over the Amazon and found that the growth of sub-10 nm particles into cloud condensation nuclei (CCN) is driven by the partitioning of semivolatile oxidation products of biogenic precursors into viscous particles. The authors modeled the impact that this enhancement of CCN has on cloud formation and found that the transition of shallow to deep convective cloud is enhanced while precipitation is suppressed. Furthermore, Shiraiwa et al.^7^ found that gas–particle interactions in viscous particles may be kinetically limited. This effect would also increase ice nucleation and enable regional transport of pollutants that are trapped in the particles.^77^ Thus, to accurately model cloud formation and chemical exposure, air quality models may need to consider effects of high viscosity and slow bulk diffusion.

## Supplementary Material



## Notes

Moving-boundary algorithm, adsorption and desorption rate coefficients, chemical mechanism, optimized parameters and MCGA, model sensitivity to parameters, comparison of the *D*_b_ – *C** relationship with data, and related data illustrations and schematics (PDF)

## ⊥

Department of Mechanical Engineering, 613 University of Colorado Boulder, Boulder, Colorado 80309, United States of America

## #

Division of Engineering and Applied Science, California Institute of Technology, Pasadena, California 91125, United States of America
