# Supplementary material for: Multiphase Chemistry and Phase State Explain Nonlinear Effects in the Formation and Evaporation of SOA from Mixed Monoterpene Precursors
Source: ACS EST Air. 2026 Apr 8;3(5):1301–11. doi: 10.1021/acsestair.5c00438 (PMC13162266; doi:10.1021/acsestair.5c00438)
Supplement: Supplementary file 1 [file ea5c00438_si_001.pdf]

# Supplementary of "Multiphase chemistry and phase state explain non-linear effects in the formation and evaporation of SOA from mixed monoterpene precursors"

Hyun Gu Kang,† Masayuki Takeuchi,‡,‖ Nga L. Ng,¶,‡,§,⊥ Ulrich Pöschl,† and Thomas Berkemeier*,†

†*Multiphase Chemistry Department, Max Planck Institute for Chemistry, 55218 Mainz, Germany*

‡*School of Civil and Environmental Engineering, Georgia Institute of Technology, Atlanta, Georgia 30332, United States of America*

¶*School of Chemical and Bimolecular Engineering, Georgia Institute of Technology, Atlanta, Georgia 30332, United States of America*

§*School of Earth and Atmospheric Sciences, Georgia Institute of Technology, Atlanta, Georgia 30332, United States of America*

‖*Current address: Department of Mechanical Engineering, 613 University of Colorado Boulder, Boulder, Colorado 80309, United States of America*

⊥*Current address: Division of Engineering and Applied Science, California Institute of Technology, Pasadena, California 91125, United States of America*

E-mail: t.berkemeier@mpic.de

# 1 Moving-boundary algorithm

With the moving-boundary algorithm, the desorption flux at the surface is counteracted by a down-motion of all layer boundaries in the model, which will cause the inner-most bulk layer to merge into the adjacent layer. Effectively, the algorithm increases diffusion from the inner-most to the outer-most bulk layer when there is net desorption to keep the volume of the near-surface bulk layer constant. While this practice still results in numerical diffusion, no sudden spikes in evaporation occur (Fig. S1). We ensure that numerical diffusion has a minor influence on the calculation results by performing calculations with increased layer resolution to check for numerical convergence. This algorithm is similar to the mass redistribution algorithm adopted by Couvidat and Sartelet [1] in the Secondary Organic Aerosol Processor (SOAP) model, where the masses of the individual bulk layers are kept equal through mass transport adjustments. However, Couvidat and Sartelet [1] did not use an adaptive layer resizing algorithm like here, and the number of bulk layers was fixed. Furthermore, the moving-boundary algorithm works to keep the volume of the near-surface bulk layer constant, not those of all bulk layers.

A schematic of the moving-boundary algorithm is in Fig. S2. When there is net desorption from the particle, we calculate the molecular volume flow out of the near-surface bulk layer ($F_{\mathrm{V,out}}$, cm$^3$ s$^{-1}$) and increase the diffusion from the layer underneath to match $F_{\mathrm{V,out}}$ (Eq. S1). Here, $y_{\mathrm{low}}$ and $V_{\mathrm{m}}$ are vectors of the component molecules and their molecular volumes (cm$^3$) in a given layer. $V_{\mathrm{low}}$ is the volume of that given layer (cm$^3$), and $A_{\text{up-low}}$ is the interface area (cm$^2$) between the given layer and the one above it.

$$F_{\mathrm{V,out}} = k_{\mathrm{b,mvbd}} \times \sum (y_{\mathrm{low}} \cdot V_{\mathrm{m}}) \frac{A_{\text{up-low}}}{V_{\mathrm{low}}} \quad \text{(S1)}$$

We find the moving-boundary bulk diffusion rate coefficient ($k_{\mathrm{b,mvbd}}$, cm s$^{-1}$) for each bulk layer interface with Eq. S1. Then, we calculate the moving-boundary diffusion rate (Eq. S2, s$^{-1}$), which is applied to every underlying layer and is added to the existing bulk

diffusion rates in the ordinary differential equations.

$$\text{Rate} = k_{\text{b,mvbd}} \times y_{\text{low}} \frac{A_{\text{up-low}}}{V_{\text{low}}} \tag{S2}$$

# 2 Adsorption and desorption rate coefficients

We explicitly model the mass transport between the near-surface gas and particle phases. For the adsorption rate coefficient ($k_{\text{a},i}$, cm s$^{-1}$) of a partitioning species $i$, we use the molecular collision rate (Eq. S3) like in Berkemeier et al.[2]. We assume a unity mass accommodation coefficient[3,4] for all partitioning species, where $\omega_i$ is the mean thermal velocity (cm s$^{-1}$) of $i$.

$$k_{\text{a},i} = \alpha \frac{\omega_i}{4} \tag{S3}$$

We use the equation for the desorption rate coefficient from particles in the chamber ($k_{\text{d,chamber},i}$, cm s$^{-1}$) from Berkemeier et al.[2] (Eq. S4), where $p_{\text{vap},i}$ is the vapor pressure (atm) of $i$ at temperature $T$ (K), $N_\text{A}$ is Avogadro's number ($6.022 \times 10^{23}$ mol$^{-1}$), $R$ is the gas constant (82.057 cm$^3$ atm K$^{-1}$ mol$^{-1}$), and $[Y]_\text{b}$ is the molecular number density of the near-surface bulk layer (cm$^{-3}$).

$$k_{\text{d,chamber},i} = \alpha \frac{\omega_i}{4} \times \frac{p_{\text{vap},i} N_\text{A}}{RT[Y]_\text{b}} \tag{S4}$$

Desorbing gases are suspected to interact with the Teflon filter and surfaces in the FIGAERO.[5] We model this effect by including a resistance term to the denominator in the equation for $k_{\text{d,chamber},i}$. The resistance term ($[Y]_{\text{FIGAERO}}$) goes into the denominator (Eq. S5), where $[Y]_{\text{FIGAERO}}$ represents the desorption delay caused by interactions between the desorbing species and FIGAERO surfaces.

$$k_{\text{d,FIGAERO},i} = \alpha \frac{\omega_i}{4} \times \frac{p_{\text{vap},i} N_\text{A}}{RT([Y]_\text{b} + [Y]_{\text{FIGAERO}})} \tag{S5}$$

# 3 Chemical mechanism

A schematic of the chemical mechanism is in Fig. S3. For simplicity, the $RO_2$ terminate to partitioning species or go down the RO pathway partitioning species that form the VBS and dimers. For both $\alpha$-pinene and limonene, the gas-phase reaction of $RO_2$ + $RO_2$ forms either an alkoxy radical (RO) or dimers (ROOR), where the ROOR branches into three logarithmically-spaced volatility bins. The $RO_2 + RO_2 \rightarrow$ ROOR branching is shown as $b_{\mathrm{dimer}}$ in Table. S1).

The initial RO either terminates into VBS monomers or isomerizes and reacts with $O_2$ to form secondary $RO_2$, whose termination products have $C^*_{298}$ that are two orders of magnitude lower. This reduction in $C^*_{298}$ is consistent with the addition of an –OH group onto a carbon backbone.[6] The fit RO termination branching is $b_{\mathrm{term.}}$ in Table. S1. The RO termination reaction produces a VBS monomer and either $NO_2$ or $HO_2$, following the fate of NAPINBO in the MCM, and this branching is fit ($b_{\mathrm{NO2\ loss}}$ in Table. S1).

As limonene possesses two double bonds, we assume the initial oxidation product retains a double bond and is highly volatile. This intermediate is consistent with limonaldehyde in the MCM, and limonone + $NO_3$ experiments report high yields of limonaldehyde.[7–10] The $C^*_{298}$ of this double-bond intermediate is optimized for, and the intermediate reacts with $NO_3$ to form $RO_2$ and subsequent products.

# 4 Optimized parameters and MCGA

We use the Monte Carlo Genetic Algorithm (MCGA)[11] to optimize the model parameters on the RAVEN supercomputer, which we then locally optimize the result with the SIMPLEX method.[12] For MCGA, we would randomly generate parameter sets on 288 CPUs, sampling 80 from each CPU. Then, each CPU would take 6 of the parameter sets and 1 randomly generated one to form a subpopulation. The genetic algorithm would pull 5 parameter sets from this subpopulation and optimize over 5 generations to find 1 best fit. The best fits

across all CPUs would be pooled, and the genetic algorithm repeats until 30 generations is reached.

As the multilayer model is quite slow, we optimize the LIM and APN scenarios separately and combine the parameter sets to optimize the parameters for the heterogeneous precursor reactions. Table. S1. summarizes the optimized values in the limonene and $\alpha$-pinene mechanisms, and Table. S2 has the values for the heterogeneous species reactions and diffusivity parameters.

# 5 Model sensitivity to parameters

To address the sensitivity of the model to optimized parameters, we provide model results after changing model input parameters individually by ±25 %. For the monomer volatility distribution, we divide the monomer volatility bins into three groups ($C^*_{298} < 10^{-1}$, $10^{-1} \leq C^*_{298} \leq 10^1$, $C^*_{298} > 10^1$ µg m$^{-3}$) and make the adjustment to each group for simplicity.

We find that the model is sensitive to the monomer volatility distributions (Fig. S10), the composition-dependent bulk diffusivity parameters (Fig. S11), the $NO_3$-aging rate coefficient ($k_{\text{aging}}$, Fig. S12), oligomerization reaction parameters (Fig. S13), and $RO_{2,\text{apn}}$ + $RO_{2,\text{lim}}$ reaction parameters (Fig. S14). In the case of the pure α-pinene experiment (APN), the model is sensitive to $k_{\text{aging}}$, while the model did not exhibit a high sensitivity for the pure limonene experiment (LIM). Given the lower bulk oligomer content from the best fit, the bulk mass is not sensitive to adjustments in $k_{\text{fwd,olig}}$ or $k_{\text{rev,olig}}$, but the thermogram models are sensitive to the energies of activation ($E_{\text{a,fwd}}$ and $E_{\text{a,rev}}$) as oligomer decomposition is sensitive to these parameters at higher temperatures. For the MIX experiment, the model outcomes are sensitive to the $RO_{2,\text{apn}}$ + $RO_{2,\text{lim}}$ cross-reaction parameters, while for the SEQ experiment it is not due to the temporal separation of $RO_{2,\text{apn}}$ and $RO_{2,\text{lim}}$ formation.

# 6 Comparison of $D_\mathrm{b} - C^*$ relationship with data

To compare the $D_\mathrm{b}$–$C^*$ relationship in this model with data, we use the data summarized by Li et al.[13]. The authors used the MPBPWIN model in the Estimation Programs Interface (EPI Suite) and the Boyer–Kauzmann rule to calculate the melting point, glass transition temperature ($T_\mathrm{g}$, K), and $C^*$ (µg m$^{-3}$) of organic compounds. We convert the $T_\mathrm{g}$ to viscosity ($\eta$, Pa s) via the Vogel–Tammann–Fulcher equation (Eqs. S6 and S7) at 298 K[13]. Here, $T_0$ is the Vogel temperature (K), and $D$ is the unitless fragility parameter, assumed to be 10.

$$T_0 = \frac{39.17 \times T_0}{D + 39.17} \tag{S6}$$

$$log_{10}(\eta) = -5 + 0.434 \times \frac{T_0 \times D}{T - T_0} \tag{S7}$$

We convert the $D_\mathrm{b,298}$ values used by KM3C to viscosities $\eta$ with the Stokes Einstein equation (Eq. S8), where $k_\mathrm{b}$ is the Boltzmann constant, $T$ is the temperature, and $r_\mathrm{m}$ is the molecular radius.

$$D_\mathrm{b} = \frac{k_\mathrm{b} T}{6 \pi r_\mathrm{m} \eta} \tag{S8}$$

Results of this calculation are shown in Fig. S15, showing that the $D_\mathrm{b}$–$C^*$ relationship employed in this study overestimates the viscosity estimates from MPBPWIN at high $C^*$ values. However, these high $C^*$ values are outside the range of the volatility distribution in this study and irrelevant for condensation of SOA. At lower $C^*$ values, values from the $D_\mathrm{b}$–$C^{**}$ relationship fall generally within the range of the data. Unfortunately, the MPBPWIN dataset is sparse and scatters immensely for these lower $C^*$ values.

Table S1: Optimized values for each scenario. The values assigned to the VBS bins are braching ratios for each bin. $\alpha$-pinene does not have a double bond intermediate in the mechanism. LB and UB refer to the lower and upper bounds set during the optimization. Branching ratios across the VBS bins for the monomers and the dimers are normalized to 1 respectively.

| | Limonene | $\alpha$-pinene | LB | UB |
|---|---|---|---|---|
| $C^*_{298} = 10^{-4}$ (µg m$^{-3}$) | 0.280 | 0.002 | Norm. | Norm. |
| $C^*_{298} = 10^{-3}$ (µg m$^{-3}$) | 0.003 | 0.004 | | |
| $C^*_{298} = 10^{-2}$ (µg m$^{-3}$) | 0.016 | 0.006 | | |
| $C^*_{298} = 10^{-1}$ (µg m$^{-3}$) | 0.219 | 0.010 | | |
| $C^*_{298} = 10^{0}$ (µg m$^{-3}$) | 0.100 | 0.025 | | |
| $C^*_{298} = 10^{1}$ (µg m$^{-3}$) | 0.001 | 0.040 | | |
| $C^*_{298} = 10^{2}$ (µg m$^{-3}$) | 0.002 | 0.039 | | |
| $C^*_{298} = 10^{3}$ (µg m$^{-3}$) | 0.379 | 0.013 | | |
| $C^*_{298} = \infty$ (µg m$^{-3}$) | 0.000 | 0.860 | | |
| Dimer bin 1 | 0.327 | 0.562 | Norm. | Norm. |
| Dimer bin 2 | 0.392 | 0.100 | | |
| Dimer bin 3 | 0.281 | 0.338 | | |
| Intermed. $C^*_{298}$ (µg m$^{-3}$) | 4640 | N/A | $10^3$ | $10^5$ |
| Dimer $C^*_{298}$ (µg m$^{-3}$) | $5.16 \times 10^{-5}$ | $1.00 \times 10^{-5}$ | $10^{-6}$ | $10^{-3}$ |
| $b_{\text{dimer}}$ | 0.0972 | 0.0144 | 0.01 | 0.1 |
| $b_{\text{NO2 loss}}$ | 0.4472 | 0.474 | 0.2 | 1 |
| $b_{\text{term.}}$ | 0.6797 | 0.937 | 0.2 | 1 |
| $k_{\text{fwd,olig}}$ (s$^{-1}$) | $3.00 \times 10^{-6}$ | $1.11 \times 10^{-5}$ | $1 \times 10^{-6}$ | $10^{-2}$ |
| $k_{\text{rev,olig}}$ (s$^{-1}$) | $3.53 \times 10^{-5}$ | $3.03 \times 10^{-6}$ | $1 \times 10^{-6}$ | $10^{-2}$ |
| $E_{\text{a,fwd}}$ (kJ mol$^{-1}$) | 98.6 | 55.0 | 30 | 150 |
| $E_{\text{a,rev}}$ (kJ mol$^{-1}$) | 44.2 | 42.1 | 30 | 150 |
| $k_{\text{aging}}$ (cm$^3$ s$^{-1}$) | $6.51 \times 10^{-15}$ | $2.53 \times 10^{-15}$ | $10^{-17}$ | $10^{-14}$ |

Table S2: Optimized values for heterogeneous reactions and $D_{\mathrm{b}}$. The slope ($m$) and intercept ($b$) are used in Eq. 4 to correlate $D_{\mathrm{b},298}$ with $C^*_{298}$. Since particle-phase oligomers do not partition in the model, we assume that they have a self-$D_{\mathrm{b}}$ that is a multiple of that of the $C^*_{298} = 10^{-4}$ µg m$^{-3}$ monomer. The $b_{\mathrm{dimer,het}}$, $C^*_{298}$ of ROOR$_{\mathrm{het}}$, $E_{\mathrm{a,fwd,het}}$ and $E_{\mathrm{a,rev,het}}$ are scaled between the values for $\alpha$-pinene and limonene, and so the LB and UB are set to those values.

| | | LB | UB |
|---|---|---|---|
| $b_{\mathrm{dimer,het}}$ | 0.0970 | 0.0144 | 0.0972 |
| $k_{\mathrm{RO2+RO2,het}}$ | $7.18 \times 10^{-13}$ | $9.2 \times 10^{-15}$ | $9.2 \times 10^{-13}$ |
| ROOR$_{\mathrm{het}}$ $C^*_{298}$ (µg m$^{-3}$) | $1.19 \times 10^{-5}$ | $1.00 \times 10^{-5}$ | $5.16 \times 10^{-5}$ |
| $k_{\mathrm{fwd,olig,het}}$ (s$^{-1}$) | $9.52 \times 10^{-6}$ | $6.00 \times 10^{-7}$ | $5.55 \times 10^{-5}$ |
| $k_{\mathrm{rev,olig,het}}$ (s$^{-1}$) | $8.08 \times 10^{-7}$ | $6.06 \times 10^{-7}$ | $1.77 \times 10^{-4}$ |
| $E_{\mathrm{a,fwd,het}}$ (kJ mol$^{-1}$) | 98.4 | 55.0 | 98.6 |
| $E_{\mathrm{a,rev,het}}$ (kJ mol$^{-1}$) | 43.1 | 42.1 | 44.2 |
| Slope ($m$) | 0.761 | 0.4 | 1 |
| Intercept ($b$) | 14.9 | 13 | 16 |
| $D_{\mathrm{b}}$ multiple for olig. | $3.91 \times 10^{-3}$ | $10^{-4}$ | 1 |
| $\Delta H^{\mathrm{dif}}$ (kJ mol$^{-1}$) | 150 | 50 | 200 |
| $[Y]_{\mathrm{FIGAERO}}$ (cm$^{-3}$) | $9.54 \times 10^{24}$ | $10^{21}$ | $10^{26}$ |

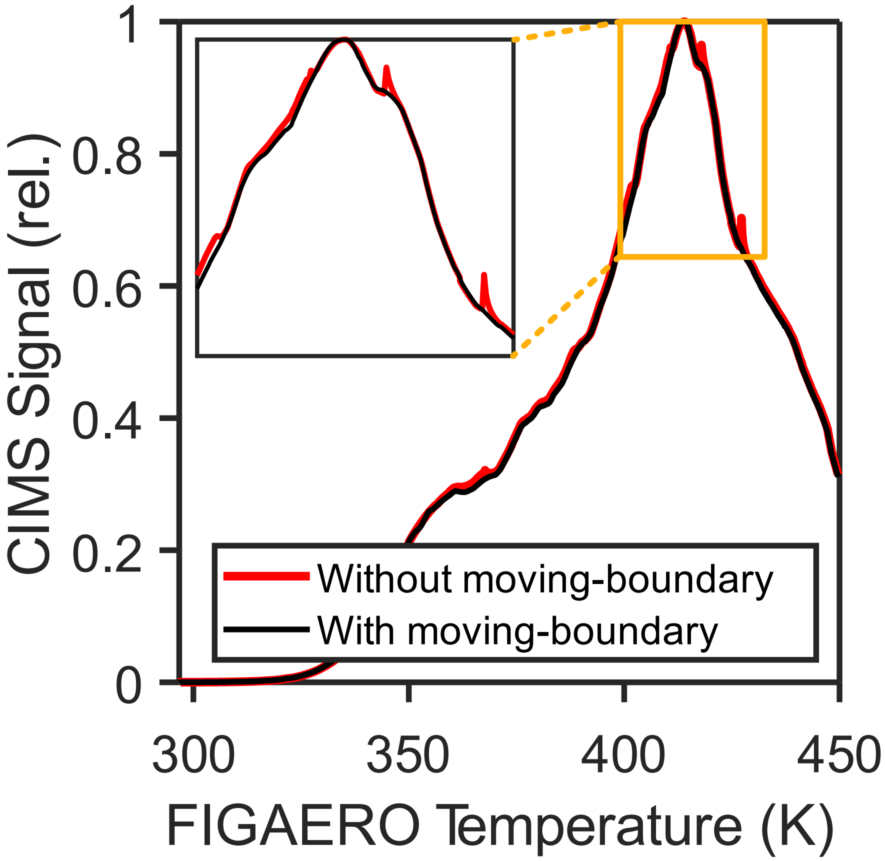


Figure S1: Smoothing of model outputs with the moving-boundary algorithm on an example FIGAERO thermogram. The black and red lines show the model results with and without the moving-boundary algorithm, and the "spikes" that appear in the red line are caused by numerical diffusion as layers merge.

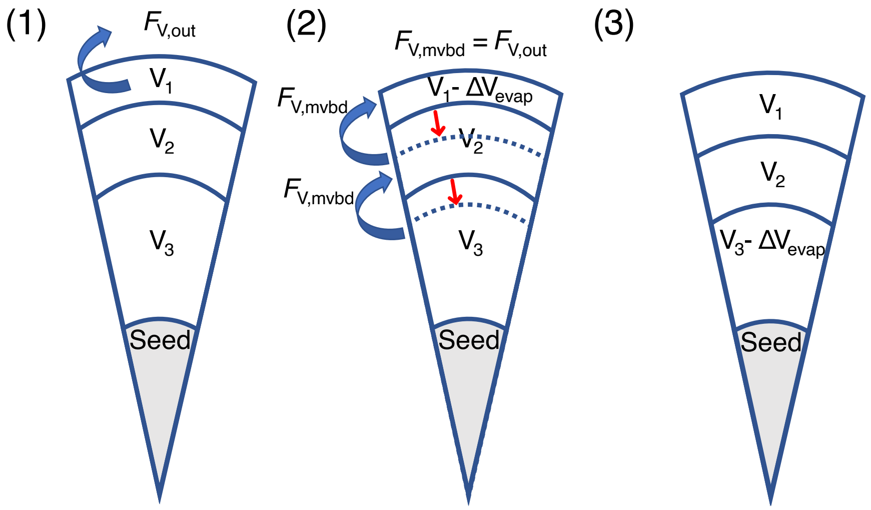


Figure S2: Illustration of the moving-boundary algorithm. When there is a positive net volume leaving the near-surface bulk layer ($\Delta V_{evap}$), diffusion from the lower layers towards the surface is increased to keep the volume of the near-surface bulk layer constant. The red arrows show the resulting shift in the bulk layer boundaries.

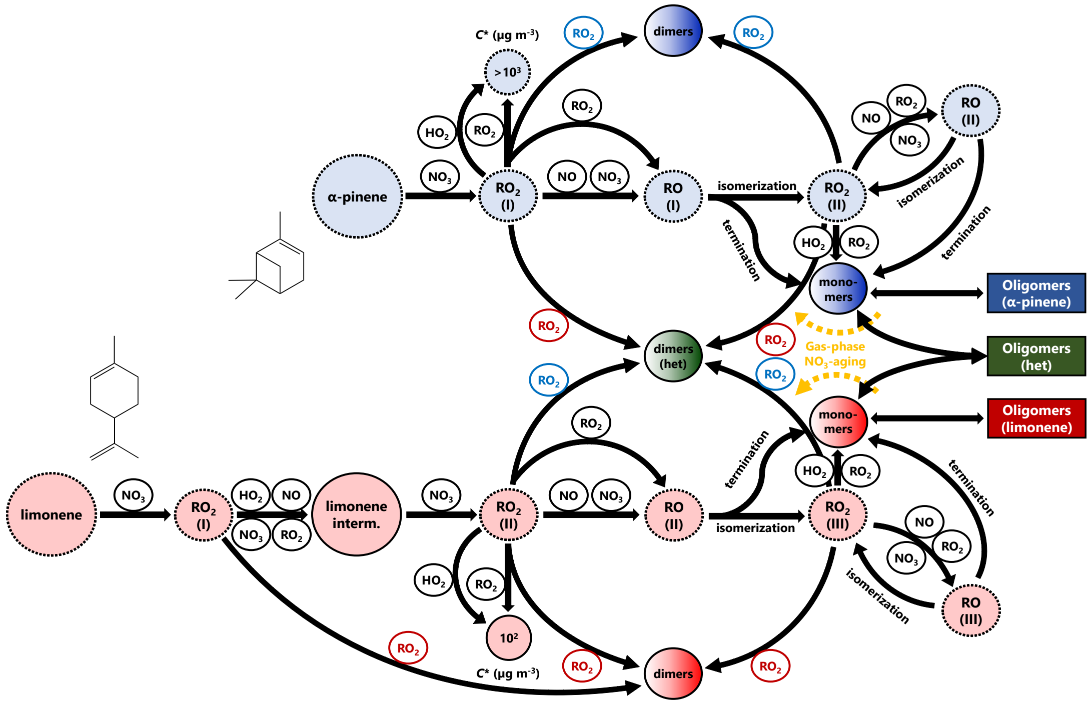


Figure S3: Mechanism schematic. Limonene follows a two-step $RO_2$ process as the molecule has two double bonds. The mechanism includes reactions where $\alpha$-pinene and limonene products react to form heterogeneous species.

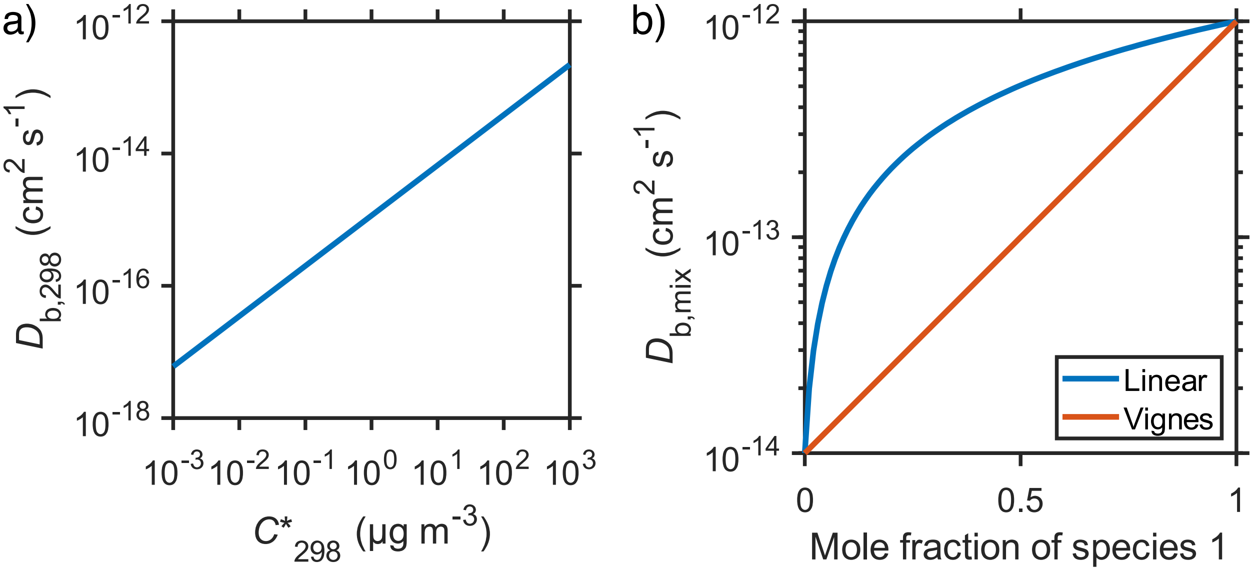


Figure S4: (a) Relationship between $C^*_{298}$ and $D_{b,298}$. (b) Illustration of Vignes and linear mixing rules with an example two species mixture. Species 1 and 2 have self $D_b$ of $1 \times 10^{-12}$ and $10^{-14}$ cm$^2$ s$^{-1}$ respectively.

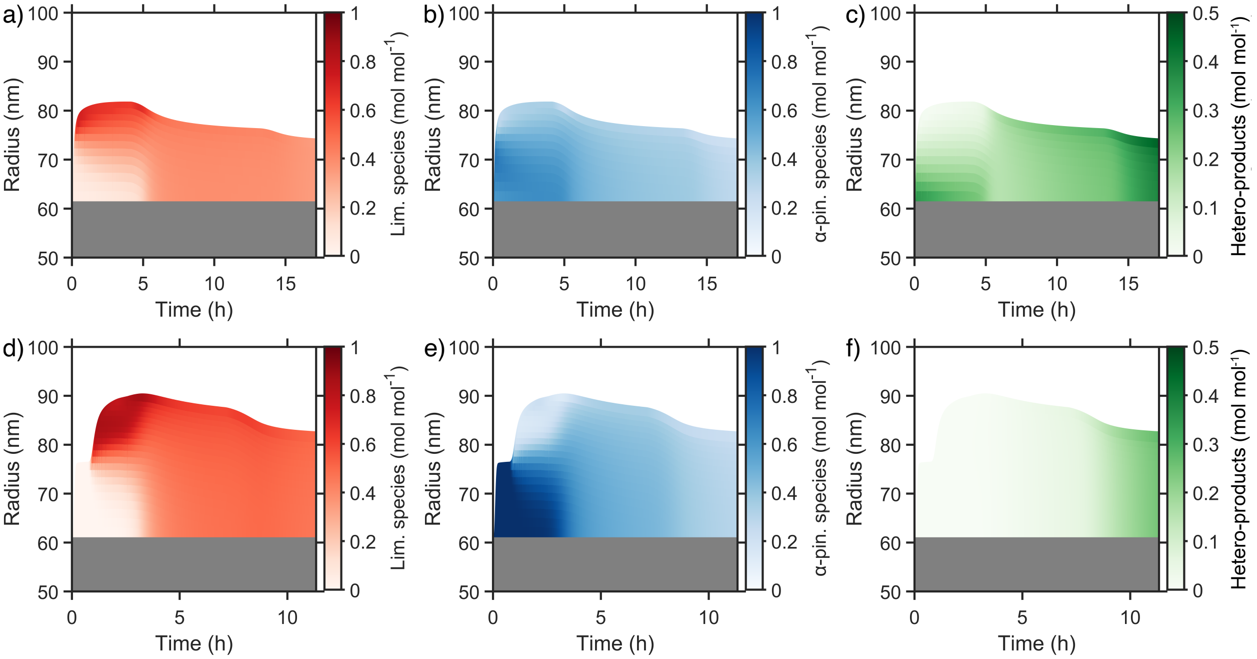


Figure S5: Limonene, $\alpha$-pinene, and heterogeneous species molar fractions in each layer for (a,b,c) MIX and (d,e,f) SEQ.

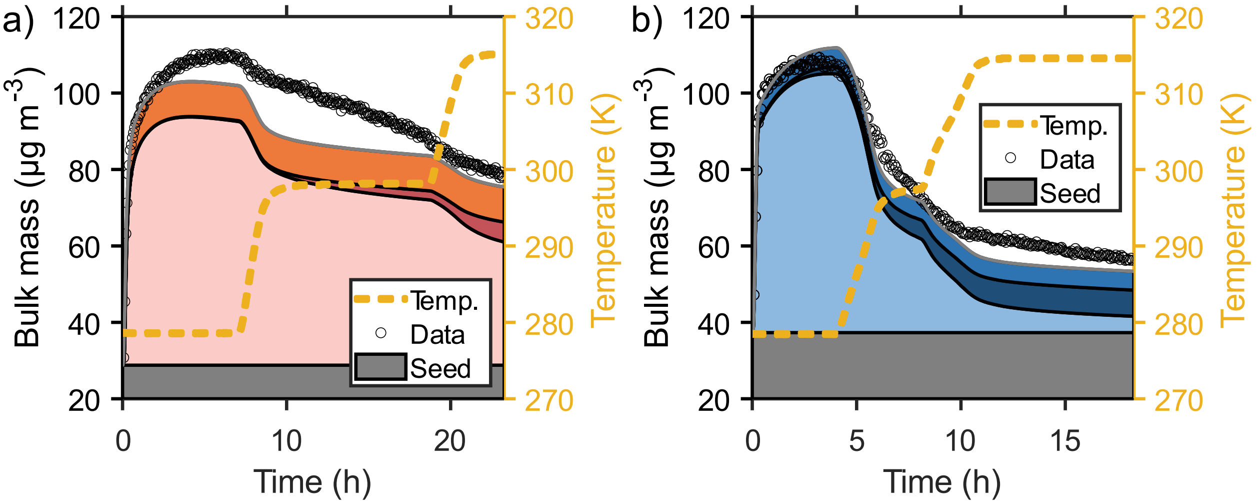


Figure S6: (a) LIM and (b) APN results from a monolayer bulk model while using fit parameters from the multilayer bulk model.

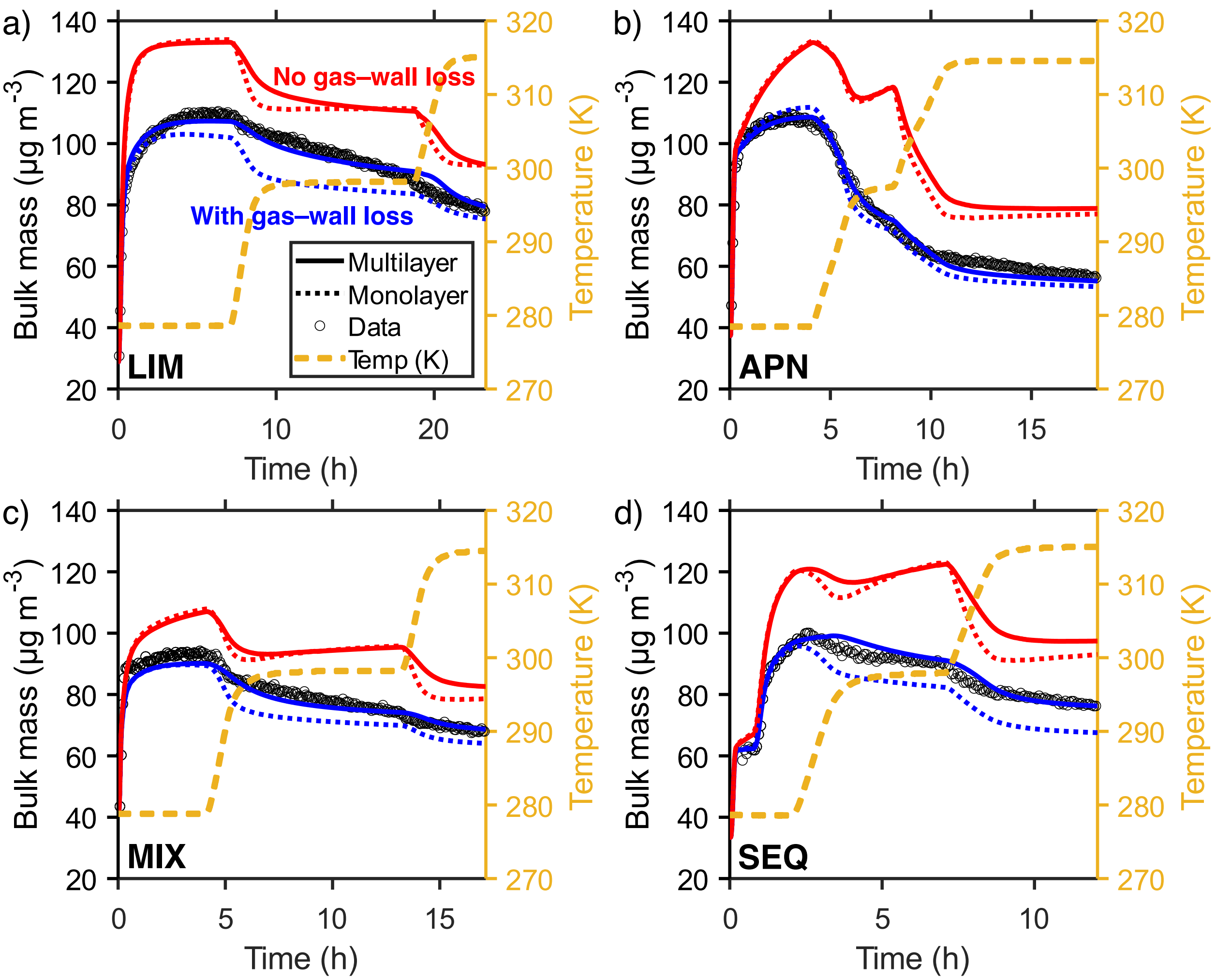


Figure S7: Multilayer (solid line) and monolayer (dotted line) model results for (a) LIM, (b) APN, (c) MIX, and (d) SEQ. Blue and red lines are results with and without gas-wall loss. The increase in SOA formation in APN and SEQ with the monolayer model come from the $NO_3$-aging of semi-volatiles evaporating with the increase in chamber temperature.

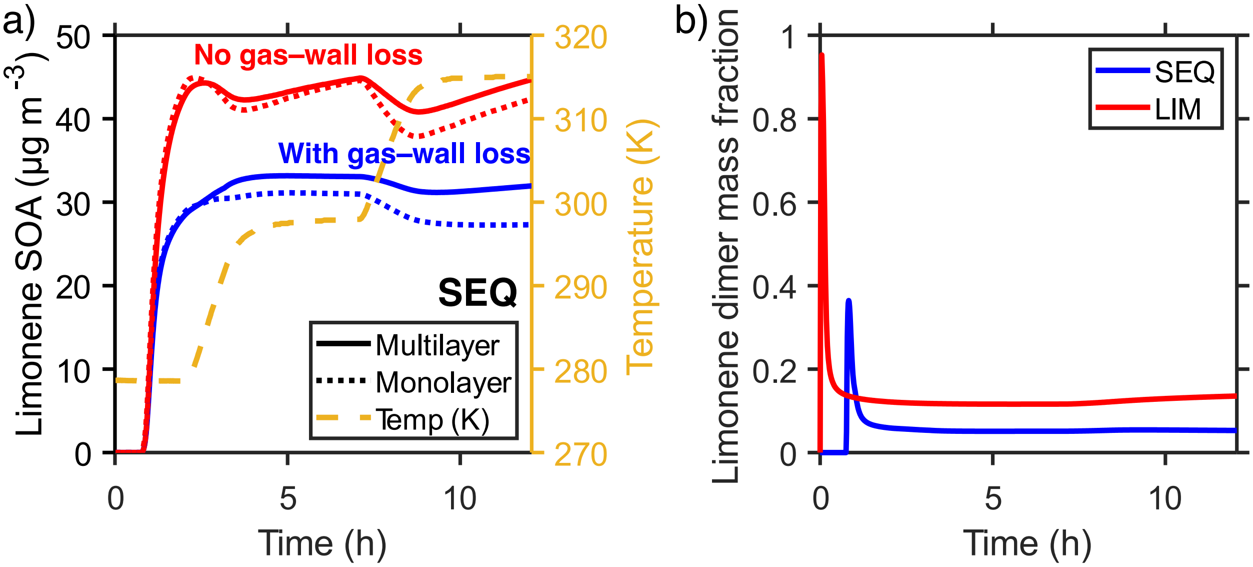


Figure S8: (a) Multilayer (solid line) and monolayer (dotted line) model results of limonene SOA in SEQ, and (b) limonene dimer fraction of limonene SOA by mass in SEQ (blue) and LIM (red). Here, the model limonene SOA and dimer masses include the limonene portion of heterogeneous species.

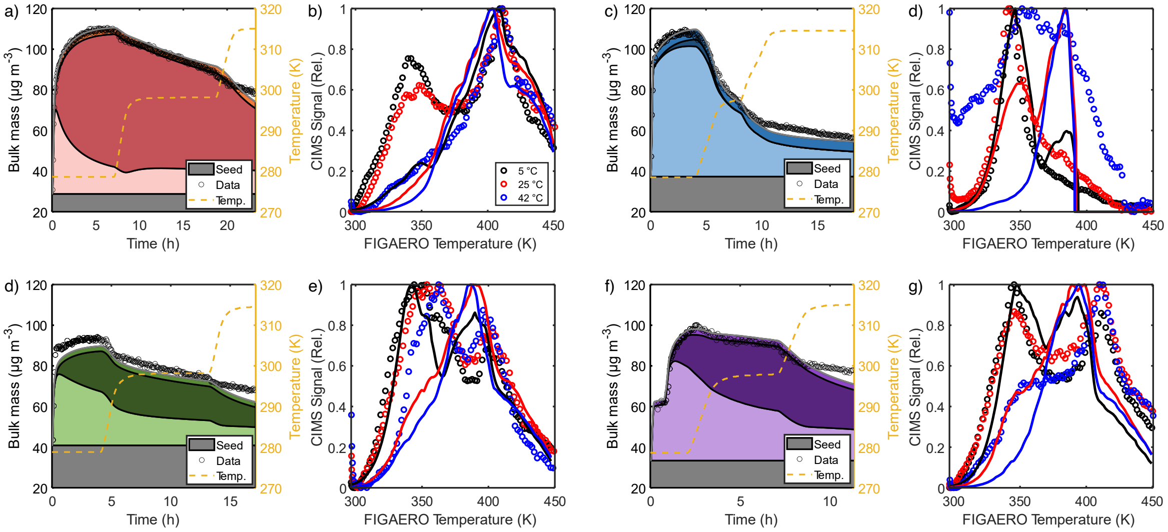


Figure S9: Optimized monolayer bulk model results of the chamber and FIGAERO scenarios for (a,b) LIM, (c,d) APN, (d,e) MIX, and (f,g) SEQ. While the model fits well with the SOA formation and evaporation in the chamber, the first peak that appears around 340 K in the LIM thermograms are not captured by the model. The first peak in the MIX and SEQ are attributed to the $\alpha$-pinene species.

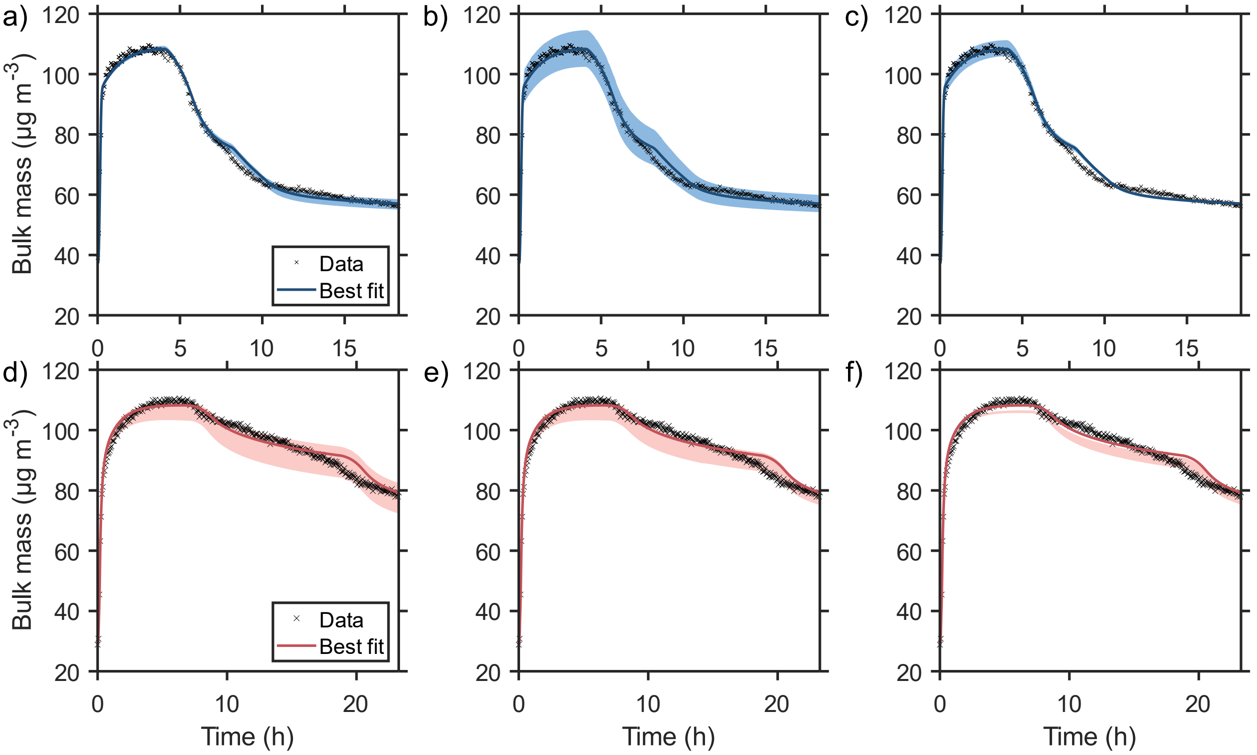


Figure S10: Model sensitivity to the monomer VBS distribution for the APN (a, b, c) and LIM chamber experiments (d, e, f). Each column shows a ±25 % adjustment to the volatility distribution $C^*_{298} < 10^{-1}$ (a, d), $10^{-1} \leq C^*_{298} \leq 10^1$ (b, e), and $C^*_{298} > 10^1$ µg m$^{-3}$ (c, f). The dark line shows the best fit result, and the shaded area shows the range of model outcomes when the parameters are adjusted within ±25 %.

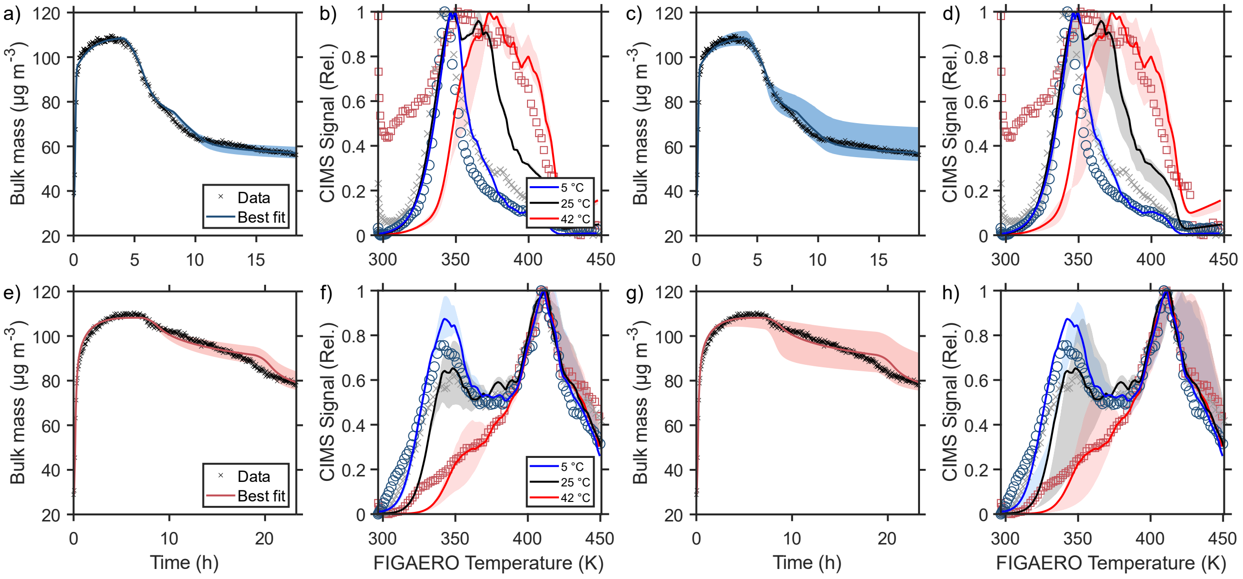


Figure S11: Chamber bulk mass and FIGAERO-CIMS thermogram model sensitivity to (a,b,e,f) the slope ($m$) and (c,d,g,h) intercept ($b$) in Eq. 4 for (a,b,c,d) APN and (e,f,g,h) LIM. The dark line shows the best fit result, and the shaded area shows the range of model outcomes when the parameter is adjusted within ±25 %.

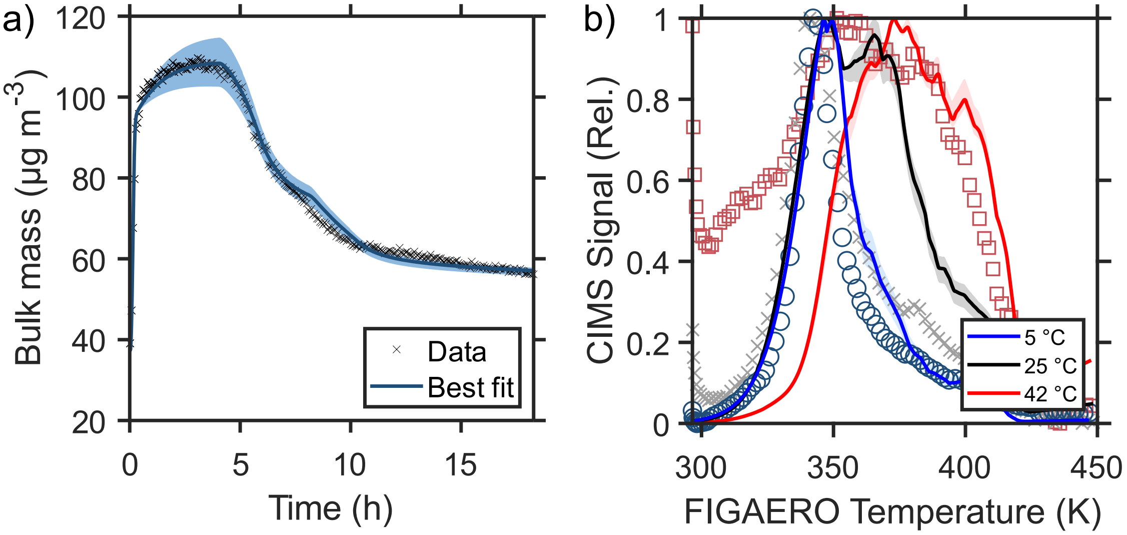


Figure S12: Model sensitivity to the $NO_3$-aging rate coefficient ($k_{\text{aging}}$) in the APN experiment. The model suggests that a higher kaging results in higher initial SOA growth. The dark line shows the best fit result, and the shaded area shows the range of model outcomes when the parameter is adjusted within ±25 %.

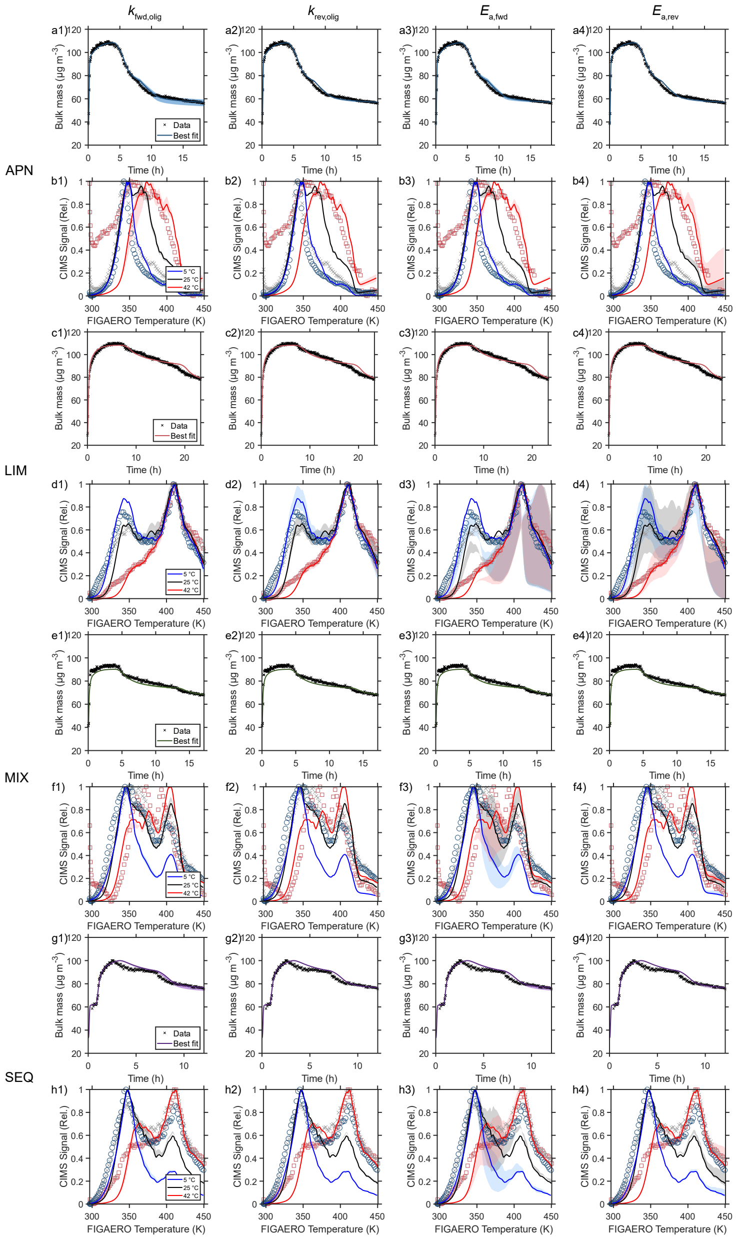

Figure S13: Model sensitivity to (1) $k_{\mathrm{fwd,olig}}$, (2) $k_{\mathrm{rev,olig}}$, (3) $E_{\mathrm{a,fwd}}$, and (4) $E_{\mathrm{a,rev}}$ in the APN (a, b), LIM (c, d), MIX (e,f), and SEQ (g, h) experiments. While the simulated SOA masses in the chamber are not very sensitive due to the slow $k_{\mathrm{fwd,olig}}$ resulting from the optimization, the FIGAERO-CIMS thermogram results are sensitive to the energies of activation of the forward and reverse oligomerization reactions.

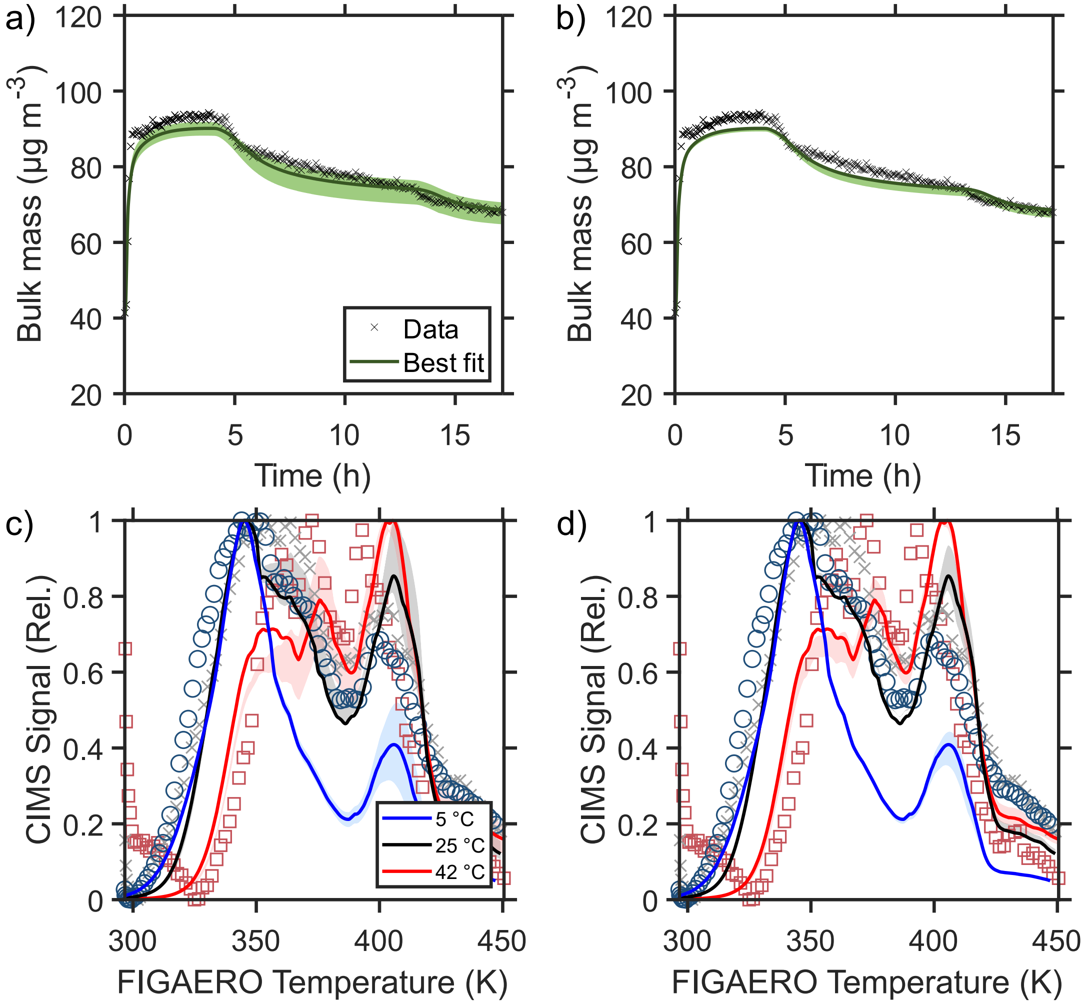


Figure S14: Model sensitivity to the branching ratio ($b_{\text{dimer,het}}$; a, c) and reaction rate coefficient (b, d) of the $RO_{2,\text{apn}} + RO_{2,\text{lim}} \rightarrow ROOR_{\text{het}}$ reaction in the MIX experiment. Higher $b_{\text{dimer,het}}$ leads to higher initial SOA growth due to rapid heterodimer formation.

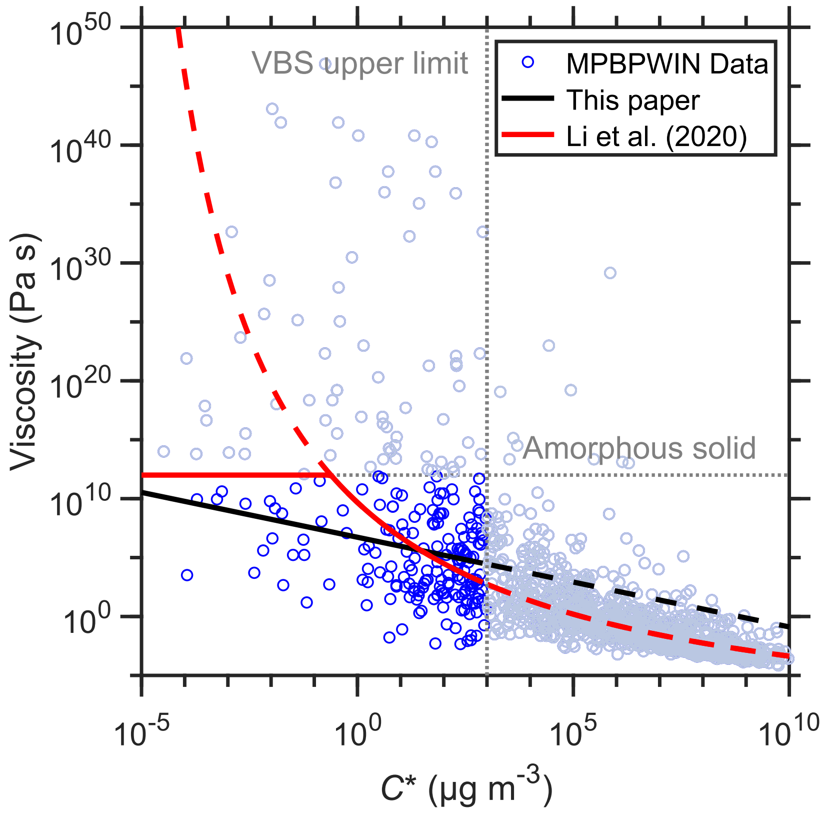


Figure S15: Comparison of $D_b$–$C^*$ relationship in KM3C when translated to viscosity values (solid black line) with MPBPWIN model data (markers) and the viscosity–$C^*$ relationship (solid red line) proposed by Li et al.[13]. Li et al.[13] also propose an SOA viscosity upper limit corresponding to viscosities of amorphous solids. The dotted black and red lines show the translated $D_b$–$C^*$ relationship and the viscosity–$C^*$ relationship beyond the VBS range in this study and viscosity limits. Within the VBS and viscosity limits in this study, the viscosities from the $D_b$–$C^*$ relationship is within the range of values predicted by MPBPWIN.

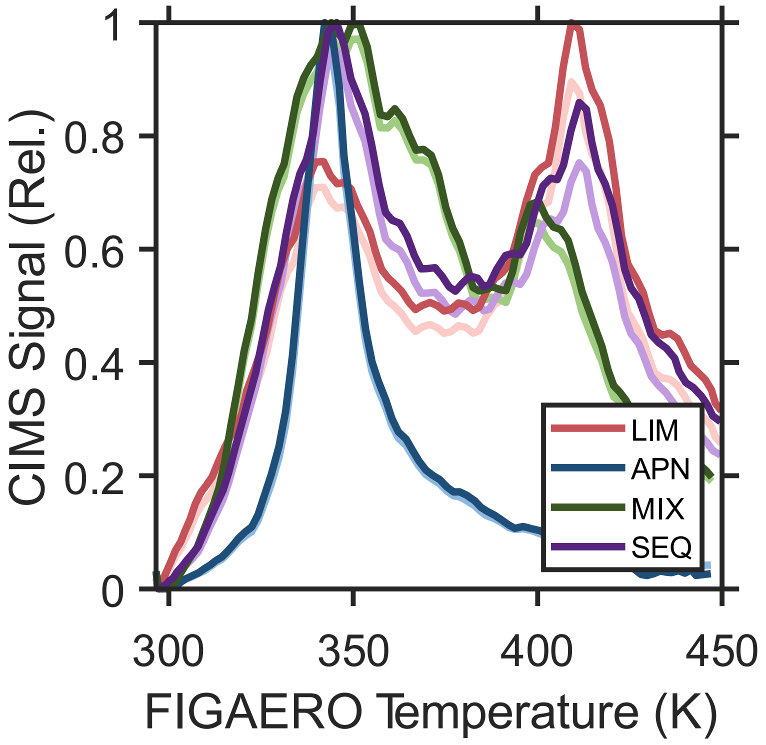


Figure S16: SOA thermograms measured with the FIGAERO-CIMS for each experiment, where dark and light lines represent all ions and the CHON ions, respectively. Across experiments, a large fraction ($> 80$ %) of detected ions contain nitrogen, suggesting that most are organonitrates.

# References


(1) Couvidat, F.; Sartelet, K. The Secondary Organic Aerosol Processor (SOAP v1.0) model: a unified model with different ranges of complexity based on the molecular surrogate approach. *Geosci. Model Dev.* **2015**, *8*, 1111–1138.

(2) Berkemeier, T.; Takeuchi, M.; Eris, G.; Ng, N. L. Kinetic modeling of formation and evaporation of secondary organic aerosol from $NO_3$ oxidation of pure and mixed monoterpenes. *Atmos. Chem. Phys.* **2020**, *20*, 15513–15535.

(3) Julin, J.; Winkler, P. M.; Donahue, N. M.; Wagner, P. E.; Riipinen, I. Near-Unity Mass Accommodation Coefficient of Organic Molecules of Varying Structure. *Environ. Sci. Technol.* **2014**, *48*, 12083–12089.

(4) Liu, X.; Day, D. A.; Krechmer, J. E.; Brown, W.; Peng, Z.; Ziemann, P. J.; Jimenez, J. L. Direct measurements of semi-volatile organic compound dynamics show near-unity mass accommodation coefficients for diverse aerosols. *Commun. Chem.* **2019**, *2*, 98.

(5) Schobesberger, S.; D'Ambro, E. L.; Lopez-Hilfiker, F. D.; Mohr, C.; Thornton, J. A. A model framework to retrieve thermodynamic and kinetic properties of organic aerosol from composition-resolved thermal desorption measurements. *Atmos. Chem. Phys.* **2018**, *18*, 14757–14785.

(6) Pankow, J. F.; Asher, W. E. SIMPOL.1: a simple group contribution method for predicting vapor pressures and enthalpies of vaporization of multifunctional organic compounds. *Atmos. Chem. Phys.* **2008**, *8*, 2773–2796.

(7) Hallquist, M.; Wängberg, I.; Ljungström, E.; Barnes, I.; Becker, K.-H. Aerosol and Product Yields from $NO_3$ Radical-Initiated Oxidation of Selected Monoterpenes. *Environ. Sci. Technol.* **1999**, *33*, 553–559.

(8) Spittler, M.; Barnes, I.; Bejan, I.; Brockmann, K.; Benter, T.; Wirtz, K. Reactions of $NO_3$ radicals with limonene and α-pinene: Product and SOA formation. *Atmos. Environ.* **2006**, *40*, 116–127.

(9) Fry, J. L.; Kiendler-Scharr, A.; Rollins, A. W.; Brauers, T.; Brown, S. S.; Dorn, H.-P.; Dubé, W. P.; Fuchs, H.; Mensah, A.; Rohrer, F.; Tillmann, R.; Wahner, A.; Wooldridge, P. J.; Cohen, R. C. SOA from limonene: role of $NO_3$ in its generation and degradation. *Atmos. Chem. Phys.* **2011**, *11*, 3879–3894.

(10) Mayorga, R.; Xia, Y.; Zhao, Z.; Long, B.; Zhang, H. Peroxy Radical Autoxidation and Sequential Oxidation in Organic Nitrate Formation during Limonene Nighttime Oxidation. *Environ. Sci. Technol.* **2022**, *56*, 15337–15346.

(11) Berkemeier, T.; Ammann, M.; Krieger, U. K.; Peter, T.; Spichtinger, P.; Pöschl, U.; Shiraiwa, M.; Huisman, A. J. Technical note: Monte Carlo genetic algorithm (MCGA) for model analysis of multiphase chemical kinetics to determine transport and reaction rate coefficients using multiple experimental data sets. *Atmos. Chem. Phys.* **2017**, *17*, 8021–8029.

(12) Press, W. H., Ed. *Numerical recipes in C: the art of scientific computing*, 2nd ed.; Cambridge University Press: Cambridge ; New York, 1992.

(13) Li, Y.; Day, D. A.; Stark, H.; Jimenez, J. L.; Shiraiwa, M. Predictions of the glass transition temperature and viscosity of organic aerosols from volatility distributions. *Atmos. Chem. Phys.* **2020**, *20*, 8103–8122.
